# Preclinical Trials of Cancer Stem Cells Targeted by Metal-Based Coordination Complexes: A Systematic Review

**DOI:** 10.3390/pharmaceutics17070931

**Published:** 2025-07-18

**Authors:** Ana Caroline Mafra Bezerra, Lucas Elohim Cardoso Viana Baptista, Maria Núbia Alencar Couto, Milton Masahiko Kanashiro

**Affiliations:** Center for Biosciences and Biotechnology, Darcy Ribeiro State University of Northern Fluminense, Campos dos Goytacazes 28013-602, Brazil; bezerra.bio.mp.mac@pq.uenf.br (A.C.M.B.); lucas.elohim2010@gmail.com (L.E.C.V.B.); nubia@uenf.br (M.N.A.C.)

**Keywords:** cancer stem cell, coordination complex, cancer treatment, systematic review

## Abstract

**Background/Objective**: Cancer stem cells (CSCs) are a self-renewing subpopulation within tumors that contribute to heterogeneity and resistance to conventional cancer therapies, including chemotherapy and radiotherapy. Despite growing interest in CSCs as therapeutic targets, effective compounds against these cells remain limited. This systematic review aims to assess the potential of metal-based coordination complexes as anti-CSC agents in preclinical models. **Methods**: A systematic literature search was conducted following the Preferred Reporting Items for Systematic Reviews and Meta-Analyses (PRISMA) guidelines. Twenty-seven original in vitro studies were included, all evaluating the cytotoxic effects of metal-based compounds on cancer cell lines enriched with CSC subpopulations. To ensure methodological rigor, all articles underwent a critical appraisal by independent reviewers who resolved discrepancies through consensus, and only studies meeting predefined quality criteria were included. **Results**: Several metal complexes, particularly copper-based compounds, demonstrated significant cytotoxicity toward CSCs, mainly through the induction of apoptosis. Breast cancer was the most frequently studied tumor type. Many studies reported modulation of CSC-related markers, including EPCAM, CD44, CD133, CD24, SOX2, KLF4, Oct4, NOTCH1, ALDH1, CXCR4, and HES1, suggesting effects on CSC maintenance pathways. Most studies were conducted in the United Kingdom and relied on in vitro models. **Conclusions**: Metal coordination complexes, especially those containing copper, show promise as therapeutic agents targeting CSCs. However, further in vivo studies and mechanistic investigations are essential to advance their translational potential.

## 1. Introduction

Cancer remains a major global public health concern. Over the past decade, its incidence has risen by 20%, and by 2030, more than 25 million new cases are projected. Cancer surveillance plays a critical role in planning, monitoring, and evaluating control measures [1]. According to the World Health Organization [2], over 35 million new cancer cases are expected by 2050, representing a 77% increase compared to the 20 million cases estimated in 2022. This rapid rise in the global cancer burden reflects not only population aging and growth but also increased exposure to risk factors, many of which are linked to socioeconomic development.

Among the multiple factors contributing to cancer progression and treatment failure, cancer stem cells (CSCs) have gained significant attention due to their central role in tumor heterogeneity, therapy resistance, recurrence, and metastasis [3,4]. Conventional chemotherapy and radiotherapy can effectively reduce the size of primary solid tumors and, when combined with surgery, eliminate the majority of cancer cells. However, CSCs often survive these treatments and can regenerate tumors, frequently displaying enhanced metastatic potential.

CSCs represent a fraction of cancer cells endowed with the abilities of self-renewal and differentiation into various progenitor cells, which sustain tumor growth in vivo. Their heterogeneity is reflected by distinct cell surface markers and their ability to undergo phenotypic plasticity, enabling them to adapt to microenvironmental changes and therapeutic stress [5,6,7]. Additionally, the clonal evolution model suggests that tumor cells initially share biological similarities but evolve into distinct clones through genetic and epigenetic alterations, resulting in differences in aggressiveness, invasiveness, and resistance to therapies [8,9,10].

The CSC hypothesis has been supported by several key observations: CSCs form a small fraction of the tumor mass but are capable of initiating tumor formation when transplanted into immunodeficient mice; they can be identified and isolated by specific surface markers; tumors originating from CSCs contain both tumorigenic and non-tumorigenic cells; and CSCs can be serially transplanted, demonstrating their self-renewal capacity [11,12]. Despite advancements in CSC research, the isolation, expansion, and in vitro study of undifferentiated CSCs, as well as the development of effective chemical agents targeting these cells, remain challenging.

Emerging features, such as phenotypic plasticity, epigenetic reprogramming, polymorphic microbiomes, and cellular senescence, have also been identified as key contributors to tumor progression [11,13,14]. Phenotypic plasticity allows CSCs to switch between epithelial and mesenchymal states, facilitating invasion and metastasis. These processes are regulated by genomic instability and epigenetic modifications, including DNA methylation and chromatin remodeling. Additionally, senescence, a state of irreversible growth arrest, can act as a barrier to malignant transformation but may also contribute to tumor progression under certain conditions.

CSCs have developed multiple mechanisms to survive environmental and therapeutic stress, including enhanced DNA repair capabilities, hyperactivation of cell cycle checkpoints, overexpression of DNA damage response proteins, increased activity of transmembrane drug efflux pumps, and the ability to enter quiescent states while maintaining basal metabolic activity [15,16]. These mechanisms are closely associated with therapy resistance, further complicating cancer treatment efforts [17,18,19].

Therefore, effective cancer treatment must target both differentiated cancer cells and CSCs. Although progress has been made in identifying specific CSC characteristics, such as surface markers and signaling pathways, no clinically approved therapies have successfully eliminated CSCs at therapeutic doses despite extensive research efforts. In recent years, metal-based compounds have emerged as a promising strategy to overcome this challenge owing to their unique physicochemical properties and diverse cytotoxic mechanisms, including induction of oxidative stress, DNA damage, and apoptosis [20,21].

Nevertheless, the limited efficacy of current anticancer metallodrugs against CSCs remains a significant obstacle, as most agents are primarily effective against differentiated tumor cells [22]. While platinum-based and other metal-containing drugs have shown restricted activity on CSCs, certain metal coordination complexes, particularly those containing copper, have demonstrated promising preclinical results. Despite substantial advancements in stem cell research, the role of CSCs in cancer recurrence and therapy resistance is not yet fully elucidated, highlighting the need for continued research. In this context, the present systematic review categorizes and analyzes preclinical studies investigating the effects of metal-based coordination compounds on CSCs, employing the Preferred Reporting Items for Systematic Reviews and Meta-Analyses (PRISMA) methodology to assess their potential therapeutic relevance across various cancer types.

Recent reviews have highlighted the therapeutic potential of metal-based coordination compounds against cancer stem cells, emphasizing their cytotoxicity, apoptotic mechanisms, and modulation of key CSC pathways, such as Wnt/β-catenin, Notch, and PI3K/Akt/mTOR [23,24,25]. These studies have provided valuable insights into the anticancer activity of metals, including copper, ruthenium, platinum, and iridium, particularly in models of breast cancer and mammosphere-forming CSCs. While these reviews offer comprehensive overviews, they often rely on narrative approaches or thematic grouping without the methodological rigor needed to minimize selection bias. In contrast, the present study introduces a systematic review conducted in accordance with PRISMA guidelines, enabling a more refined and reproducible selection of eligible studies. By applying predefined inclusion criteria and critically appraising methodological quality, this review not only consolidates preclinical data on the cytotoxic effects of coordination complexes in CSC-enriched models but also identifies gaps in selectivity and toxicity profiles. This systematic approach distinguishes it from prior reviews by enhancing analytical specificity and providing a robust framework for guiding future translational research.

## 2. Materials and Methods

This systematic review was prospectively registered in the International Prospective Register of Systematic Reviews (PROSPERO) database under the registration number [ID1066596]. The protocol followed the Preferred Reporting Items for Systematic Reviews and Meta-Analyses (PRISMA) guidelines to ensure transparency and methodological rigor.

### 2.1. Search Strategy

The systematic review followed the PRISMA guidelines [26]. The search strategy was applied to multiple electronic databases, including PubMed, Scopus, Web of Science, and Google Scholar. The primary databases (PubMed, Scopus, Web of Science) were selected because they index peer-reviewed, high-quality scientific literature relevant to biomedical and pharmaceutical sciences. Google Scholar was also included to enhance coverage by capturing gray literature, conference proceedings, and early-stage studies not yet indexed in traditional databases; however, all such records were carefully screened to ensure scientific rigor and peer-review status.

**Figure 1:**
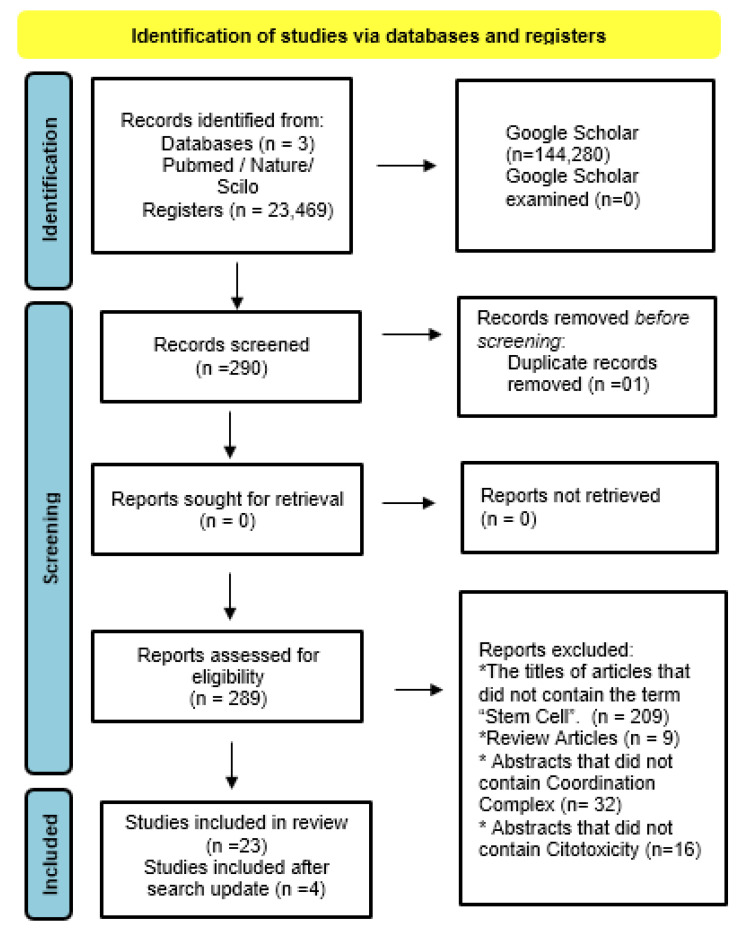
PRISMA diagram of the systematic review. The retrieval process is illustrated, covering the stages of identification, screening, eligibility assessment, and inclusion of studies. A total of 23,469 studies were initially identified through database searches. After removing duplicates, 289 records were screened by title and abstract. Subsequently, 23 full-text articles were assessed for eligibility, resulting in 27 studies included in the qualitative synthesis. In the excluded reports, articles whose abstracts did not contain the following were excluded: Titles with the term cancer stem cells present, literature review articles, abstracts that do not specify the coordination complex and that do not contain cytotoxicity assays. Note that *n* indicates the number of studies included at each stage. This diagram provides a clear overview of the study selection and reasons for exclusion throughout the process.

A bibliographic search was conducted in PubMed, Nature, Scielo, and Google Scholar between 11 December 2023 and 12 December 2023, with an update on 27 January 2025. The general breakdown of item quantities is specified in (Supplementary Table S1). Both searches used the terms “Cancer Stem Cell” and “Coordination Complex” and included various related keywords (detailed in a Supplementary Table). The keyword combinations used were [“Cancer Stem Cell” AND coordination complex OR metallocomplex] and [“Cancer Stem Cell” AND coordination complex], applied mainly in PubMed and Nature databases. This strategy identified 290 articles, with 1 duplicate excluded (see Figure 1). The search strategy aimed to be broad enough to avoid missing relevant studies while prioritizing high-quality indexed sources to ensure consistency of peer-reviewed content.

### 2.2. Study Selection and Data Extraction

The results were exported for title, abstract, and full-text screening. The inclusion criteria were (1) article titles containing the term “Stem Cell”; (2) original experimental studies involving coordination complexes; and (3) abstracts addressing stem cell characterization, coordination complexes, and cytotoxicity. Studies focusing on cell death characterization were also considered, regardless of exclusion criteria. After screening, 20 articles underwent full-text evaluation, of which 3 met all criteria and were included. The PRISMA flowchart in Figure 1 details the study identification, inclusion, and exclusion process.

The data extracted included information on stem cells and coordination complexes, such as country of origin and year of publication, experimental methods, types of coordination compounds used, stem cell characteristics, cancer cell lines studied, types and mechanisms of induced cell death, and contributions to the preclinical evaluation of metallopharmaceutical treatments targeting stem cells.

### 2.3. Assessment of Methodological Quality of Included Articles

Currently, there is no defined methodological tool for bias analysis in systematic reviews of in vitro preclinical trials. Many studies adapt existing criteria from platforms that evaluate randomized or non-randomized clinical trials, adjusting their analyses according to their individual criteria. Due to the particularities of preclinical studies, it is difficult to establish questions for critical analysis of these studies, since their quality depends on the type of experimental model used.

From this perspective, this review applied the criteria recommended for basic research in epidemiology within preclinical studies, as they were best suited for the analysis conducted [27], Explained in (Supplementary). These criteria are detailed in the Supplementary Materials and associated with Likert-scale statements for accurate analysis. Three independent reviewers (AB, LE, and MC) evaluated all selected articles to ensure consistency and quality in the analysis, aligning with the parameters established for this review. Articles that did not achieve at least 50% agreement on positive statements (“I completely agree” and/or “Agree”) were classified as low quality and excluded from further analysis (Supplementary Table S2 and Graph S1). A survey of all experiments carried out by the research of the analyzed articles was also carried out and these were grouped and presented in the Supplementary Table S3.

## 3. Results

- Systematic Review

**Figure 2:**
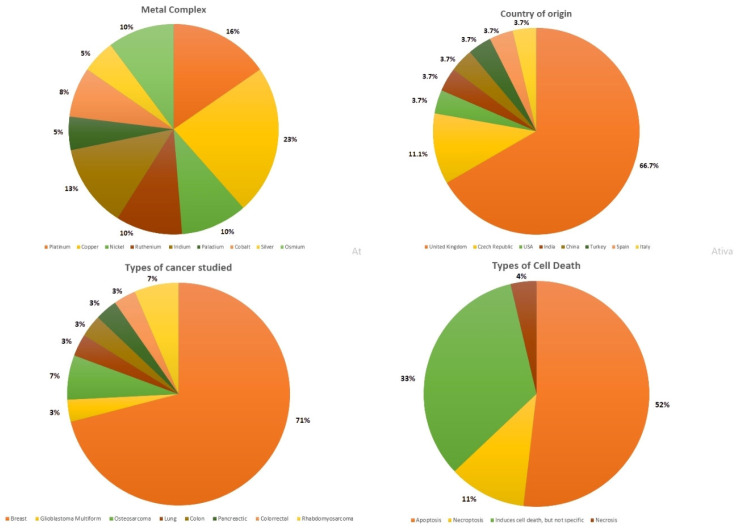
Graphical representations showcasing the percentages of general characteristics extracted from the analyzed articles. These characteristics include the country of origin of studies, the types of cancers investigated, the metal complexes examined, and the types of cell death identified in the analysis results.

The PRISMA flowchart in Figure 1 describes the process of identifying, including, and excluding studies. Article selection was made from studies conducted in the last 10 years, with 27 studies (Table 1) selected based on the PRISMA criteria, as detailed in the Supplementary Materials. The overall percentage data are provided, with corresponding illustrations in Figure 2. For experimental analyses, the MTT [3-(4,5-dimethylthiazol-2-yl)-2,5-diphenyltetrazolium bromide] cell viability assay was used in 100% of the studies to assess the cytotoxicity of metal complexes, while other techniques are described in the Supplementary Materials. All studies also explored the potential mechanisms of action of the complexes. The results were categorized into the following: Characteristics of the metal complexes: Detailed descriptions of their structural properties, chemical compositions, and relevant characteristics; Cytotoxicity in stem cells and cancer stem cells: Assessment of the effects on normal and cancer stem cells, their influence on stem cell markers, and the types of cell death analyzed. General characteristics are categorized in Table 2.

Subsequently, we assessed the biological relevance of the synthesized compounds and their effects on stem cells. All the reviewed studies employed the MTT assay to evaluate cell viability following treatment with the synthesized complexes. This approach provided insights into the cytotoxic potential of the compounds and their ability to impact stem cell populations, highlighting their therapeutic prospects in cancer research. These analyses are specified in Table 3.

### 3.1. Analysis of the Characteristics of Metal Complexes

#### 3.1.1. Platinum

Platinum (II) complexes, such as cisplatin, carboplatin, and oxaliplatin, are commonly used in clinical settings to treat different types of cancers. Considerable effort has been dedicated to elucidating their cellular and molecular mechanisms of action [56]. The primary therapeutic impact of platinum (II) agents stems from their capacity to form covalent bonds with DNA, causing structural distortions [57]. This prevents DNA replication and transcription, ultimately inducing programmed cell death. However, despite their efficacy, platinum (II) drugs present notable limitations. These include systemic toxicity and side effects arising from their inability to differentiate between rapidly dividing cancer cells and healthy proliferating cells [58]. Moreover, acquired or inherent resistance renders them ineffective against certain tissue types, increasing the likelihood of cancer recurrence [59,60].

Indeed, numerous independent in vitro and in vivo studies have demonstrated that cisplatin, carboplatin, and oxaliplatin tend to enrich rather than deplete CSCs within heterogeneous tumor populations [56]. This phenomenon is mainly attributed to elevated levels of DNA-repair-associated factors (such as BRCA1, ATR, ATM, and Chk1) and platinum-related drug efflux pumps (such as ATP-binding cassette transporters) in CSCs [61]. Despite these challenges, significant research efforts continue to increase the efficacy of platinum-based complexes, particularly in CSCs. Although progress is being made in developing such complexes that can target cancer cells and CSCs at clinically relevant concentrations, only a few platinum complexes have shown substantial promise to date.

Among these are a platinum(II) complex, trans-[PtCl_2_(2-(2-hydroxyethyl)pyridine)_2_], and three platinum(IV) complexes: cis,trans,cis-[Pt(NH_3_)_2_(OH)(cinnamic acid)Cl_2_], cis,trans,cis-[Pt(NH_3_)_2_(cinnamic acid)_2_Cl_2_], and cis,trans,cis-[Pt(NH_3_)_2_(t-cinnamate)(oleate)Cl_2_]. These compounds effectively reduced the viability of breast and rhabdomyosarcoma CSCs in vitro at micro- or submicromolar concentrations [62]. Furthermore, a synthesized platinum complex [Pt (1,5-ciclooctadieno)Cl_2_] was more potent and demonstrated impressive efficacy 56–77 times greater against CSC mammospheres compared to salinomycin, cisplatin, and carboplatin [44].

Another approach involves combining platinum complexes with other substances known for their antitumor activity, thereby enhancing their effectiveness. The design of platinum (IV) complexes offers the opportunity to conjugate biologically active ligands in axial positions. These ligands can be released concurrently with the DNA-damaging Pt (II) portion once the complexes accumulate and undergo reduction within cells. This enables them to act on various cellular targets through different mechanisms [63,64]. Therefore, these conjugates have the ability to deliver not only the molecules of the platinum component into tumor cells but also molecules of other biologically active compounds.

Two platinum (IV) cisplatin derivatives with axial cinnamate ligands were synthesized, showing antiproliferative activity at submicromolar concentrations, particularly against rhabdomyosarcoma, a challenging pediatric cancer. The study suggests that cinnamic acid, incorporated in these complexes, enters cells with platinum and may promote differentiation, enhancing the sensitivity of CSCs to platinum’s cytotoxic effects. This dual-component approach improves cellular uptake, leading to higher intracellular concentrations of both platinum and cinnamic acid. Despite these advancements, there is still a need for new platinum agents that can effectively target both bulk cancer cells and CSCs at clinically relevant concentrations [48].

#### 3.1.2. Copper

Copper (Cu), an essential trace element for the human body, plays a fundamental role in several biological metabolic processes, which are important for the support, growth and development of the organism. It also plays a role in several biological functions, such as growth, cardiovascular health, and neuroendocrine activity. It is involved in several cellular processes and is incorporated into bioactive compounds used to treat conditions like copper deficiency, inflammation, and rheumatoid arthritis [34,65]. This wide range of biological functions highlights its potential for therapeutic applications [66]. Copper’s lower toxicity compared to non-essential metals has driven research into copper-based compounds as potential alternatives to platinum-based anticancer drugs [67]. The effectiveness of copper complexes in cancer treatment depends largely on the coordinating ligands, which influence their cytotoxicity and mechanism of action [68].

The action mechanism of copper (II) complexes is primarily dictated by their coordinating ligands. Many of these complexes induce cancer cell death by promoting oxidative stress through the generation of reactive oxygen species (ROS) via Fenton-type reactions. Additional mechanisms include DNA binding, inhibition of topoisomerases I and II, and disruption of the proteasome. However, a common challenge with copper-based ROS inducers is their tendency to undergo change and release copper during the redox processes necessary for ROS production. This occurs because copper (I) and copper (II) oxidation states favor different geometrical configurations. Copper (I) typically prefers tetrahedral geometries, though it can also adopt linear and trigonal planar structures, while copper (II) centers tend to adopt square planar, trigonal pyramidal, or octahedral configurations [47,69,70].

Copper (II)–phenanthroline complexes incorporating non-steroidal anti-inflammatory drugs (NSAIDs) have been shown to selectively eliminate mammary CSCs while preserving other cell types at micro- to submicromolar concentrations [71]. The cytotoxic mechanism of these complexes on mammary CSCs involves, in part, the intracellular increase in ROS, activation of oxidative stress pathways, and caspase-dependent apoptosis [34]. Copper (II)–Schiff base complexes are known as potent ROS generators, capable of inducing apoptosis in various cancer cells through mechanisms such as DNA damage or mitochondrial dysfunction. Furthermore, it has been reported that copper (II) complexes containing vanillin–Schiff base derivatives and the NSAID naproxen increase intracellular ROS levels within a short period of exposure (six hours), leading to DNA damage, reduced COX-2 expression, and activation of caspase-dependent apoptosis [51].

A study investigating strategies to improve the effectiveness of copper coordination compounds, using copper–dithiacyclam complexes stabilized by donor pools (reserves of molecules that can donate electrons during redox reactions), such as type I electron transport proteins (e.g., plastocyanin and azurin), demonstrates that this type of design supports efficient redox cycling between copper (I) and copper (II) states without copper leaching, thanks to hard and soft ligands that stabilize both oxidation states and reduce the reorganization energy, increasing ROS generation. Thus, in the context of these complexes, electron donors play a fundamental role in facilitating the transfer of electrons between molecules or cellular components [37].

In parallel, research on copper (II) complex combinations revealed that the structure [Cu(barb-κN)(barb-κ^2^N,O)(phen-κN,N′)]·H_2_O optimized therapeutic outcomes. The combination of valproic acid with this complex significantly inhibited the proliferation of breast CSCs by inducing apoptosis and oxidative stress through ROS production. These findings suggest a promising potential for breast cancer therapy, although further clinical trials are needed to evaluate efficacy and safety [38]. Many copper complexes show cytotoxic effects against cancer cells, prompting growing interest in developing copper-based coordination compounds for pharmacological applications. The precise molecular mechanism connecting copper concentration to cancer cell activity is still under investigation.

#### 3.1.3. Nickel

Investigations into nickel-containing small molecules as potential anticancer agents have been conducted, and although few studies detail their cellular mechanisms of action, there is current research into their stem cell anticancer properties [72]. Among these, two nickel (II)-3,4,7,8-tetramethyl-1,10-phenanthroline complexes containing NSAID groups (naproxen and indomethacin) have been reported, which have demonstrated the ability to kill breast cancer cells in large quantities; furthermore, these complexes affected both breast cancer cells and breast CSCs in the micromolar range under in vitro monolayer conditions [42]. Nickel (II) complexes containing semicarbazone and thiosemicarbazone ligands demonstrate significant in vitro antiproliferative activity and are believed to exert their cytotoxic effects by causing damage to genomic DNA or inhibiting topoisomerase II [73]. Other studies have indicated that a nickel (II) pyrithione complex could effectively inhibit the proliferation of cultured tumor cells, primary cells derived from human patients with acute myeloid leukemia, as well as xenografts of chronic myeloid leukemia (K562) and lung carcinoma (A549) in nude rats [7].

Mechanistic studies have revealed that the nickel (II) complex exhibits all the hallmarks of necroptosis, such as necrosome-mediated cell membrane disruption, mitochondrial depolarization, and distinct necroptotic morphological features. Furthermore, unbiased predictive functional genetic analysis based on RNA interference (RNAi) demonstrated that the mechanism of action of the nickel (II) complex resembles that of shikonin, a genuine inducer of necroptosis [74]. Other additional mechanistic studies indicate that a series of phenanthroline nickel (II)–dithiocarbamate complexes induce necroptosis in breast CSCs through necrosome-mediated cell membrane disruption and mitochondrial membrane depolarization. Morphological changes consistent with necroptosis were also observed in the treated CSCs [75].

Studies reveal that inducing necroptosis in cancer models activates cytotoxic T cells, promoting tumor regression and protection against recurrence [76]. A nickel (II) complex with 3,4,7,8-tetramethyl-1,10-phenanthroline and flufenamic acid has shown potential as a necroptosis inducer in osteosarcoma stem cells, possibly via COX-2-mediated pathways [35]. Additionally, nickel-based complexes, such as those with N-aroyl-N′-thiohydrazide ligands, induce apoptosis through chromatin condensation and cell cycle arrest, inhibiting Dalton’s lymphoma growth and reversing immunosuppression [60].

#### 3.1.4. Iridium

Iridium (Ir) is a transition metal from the third row of the periodic table, belonging to the same group as cobalt (Co) and rhodium (Rh). It is categorized as one of the “precious metals” within the platinum-group elements. Iridium is relatively uncommon and was first discovered in 1803 as an impurity found within platinum deposits. Iridium metal is known for its inertness and resistance to corrosion [35]. Initial studies on the anticancer activity of organoiridium complexes primarily concentrated on square-planar Ir (I) complexes. This focus was due to their structural and electronic resemblance with Pt (II) anticancer complexes like cisplatin [77].

Organometallic iridium (III) complexes with pyridocarbazole have been shown to possess in vivo antiangiogenic properties in zebrafish models, along with photocytotoxicity against bulk cancer cells [78,79]. Additionally, noteworthy research has indicated that organometallic iridium (III) complexes containing electron-rich pentamethylcyclopentadienyl ligands exhibit promising potency against various types of cancer cells, with efficacy demonstrated down to submicromolar levels [77].

Certain cyclometalated iridium (III) complexes have been identified as efficient producers of photoinduced singlet oxygen in bulk cancer cells [80]. Research into the anticancer properties of iridium (III) complexes has shown that their cytotoxicity and mechanism of action are strongly influenced by the coordinating ligands [15]. These ligands also play a key role in determining the intracellular localization, with bulky, lipophilic ligands promoting mitochondrial accumulation [81]. Additionally, a study found that photoactivatable dipyridophenazine iridium (III) complexes exhibited high phototoxicity (λ exc = 420 nm) in HeLa cells, a model of human cervical cancer, even under hypoxic conditions. The study further revealed that in HeLa cells treated with these complexes, the photochemical mechanism shifted from Type II to Type I, thereby improving photodynamic therapy (PDT) efficacy in hypoxic conditions. This enhancement addresses a limitation of PDT, with the complexes showing effectiveness at submicromolar concentrations [22].

Iridium (III) complexes containing 1,10-phenanthroline demonstrated equal potency (in the micromolar range) against both bulk breast cancer cells and breast CSCs, suggesting their potential to eliminate heterogeneous breast cancer populations with a single dose. Additionally, mechanistic studies revealed that the complex inhibits cytochrome c oxidase activity, induces depolarization of the mitochondrial membrane, increases intracellular ROS, and triggers caspase-dependent apoptosis. These findings underscore the potential of mitochondrial targeting agents as anti-CSC agents and pave the way for the development of other iridium (III) complexes as active agents targeting mitochondria in CSCs [50].

#### 3.1.5. Palladium

Palladium complexes have low intrinsic solubility in aqueous solutions, which is a limiting factor in their use [56]. However, padeliporfin (TOOKAD), a bacteriochlorophyll-based palladium (II) complex, has emerged as the pioneer palladium-containing compound approved for clinical application. TOOKAD is used in PDT for the treatment of prostate cancer. In addition to TOOKAD, numerous palladium complexes have been recognized for their potential in promoting anticancer activities against bulk cancer cells, both in vitro and in vivo [6].

Other metal complexes from group 10, such as those incorporating palladium, have been widely studied for their potential as antineoplastic and antimetastatic agents [82]. Palladium coordination complexes typically display higher lability and faster ligand exchange rates (approximately 10^5^ times faster) compared to their platinum analogs. Developing highly effective anticancer agents with low systemic toxicity by leveraging palladium’s reactivity poses a significant challenge [6].

Studies show that a cationic palladium (II)–terpyridine complex with a saccharinate counteranion exhibits superior or equivalent potency against prostate CSCs compared to cisplatin and etoposide. It induces DNA damage, caspase-dependent apoptosis, and autophagy in prostate cancer cell lines and patient-derived cells, with similar findings in metastatic breast and cervical cancers [83,84,85]. A trinuclear palladium (II) complex further demonstrated enhanced inhibition of CSC spheroids compared to salinomycin by inducing DNA damage and apoptosis [45].

Other studies have shown that a monocationic square-planar palladium (II)–terpyridine complex with a saccharinate counteranion effectively eliminates prostate CSCs in vitro at micromolar concentrations [75]. Despite the palladium (II) centers being tightly bound to coordinated ligands and exhibiting solution stability, these complexes are unlikely to interact with DNA covalently. However, the research indicates that stable palladium (II) complexes in solution can induce toxicity. In CSC mammospheres, these complexes penetrate significantly, infiltrating CSC nuclei, causing genomic DNA damage, and triggering caspase-dependent apoptosis [39]. This analysis highlights the therapeutic potential of palladium-based agents and suggests their development as effective therapies against CSCs.

#### 3.1.6. Cobalt

Hypoxia-inducible factors (HIFs), including HIF1α and HIF2α, are recognized as crucial microenvironmental elements involved in regulating CSCs [17]. Consequently, there is a proposal suggesting that properly engineered hypoxia-activatable cobalt (III) prodrugs represent a promising strategy for eradicating CSCs [56]. However, a significant limitation of the Co (III) complex known for its CSC-targeting activity is its susceptibility to rapid reduction and premature detachment from monodentate-bound NSAID ligands under conditions mimicking physiological environments [62].

Nevertheless, an active Co (III)–cyclam complex has been documented in CSCs, incorporating two units of monodentate-bound NSAIDs. NSAIDs function by inhibiting cyclooxygenase (COX-1 or COX-2)-mediated prostaglandin production, which serves as an inflammatory effector [86]. COX-2, the inducible isoform, is often overexpressed in specific CSC populations and contributes functionally to their proliferation. Hence, inhibiting COX-2 proves to be an effective approach in sensitizing CSCs to cytotoxic agents [87].

The cobalt (III) complex exhibited the ability to eliminate CSCs and cancer cells at low micromolar concentrations. Specifically, cobalt (III)–polypyridyl complexes containing diflunisal were observed to release diflunisal under reducing conditions. Cell-based mechanistic investigations revealed that this complex induced DNA damage and inhibited COX-2, leading to the eradication of CSCs. The release of diflunisal likely sensitizes CSCs to the cytotoxic effects of the reduced products of the complex [49]. The cobalt (III)–cyclam complex efficiently penetrates mammary CSCs, induces DNA damage, and triggers caspase-dependent apoptosis while reducing COX-2 expression, likely due to its flufenamic acid moieties. Its capacity to induce immunogenic cell death (ICD) was evidenced by increased calreticulin (CRT) surface expression, adenosine triphosphate (ATP) and high mobility group box 1 protein (HMGB-1) release, and enhanced macrophage phagocytosis of the treated CSCs. These findings underscore the immunotherapeutic potential of cobalt complexes for CSC-targeted therapy.

#### 3.1.7. Silver

Silver serves no recognized biological function; nevertheless, the body can withstand low levels of silver without experiencing any toxic side effects [11]. Nonetheless, a wide array of structurally diverse silver compounds, including those with carboxylic acids, amino acids, nitrogen, phosphorus, and sulfur-donating ligands, have been investigated as potential antitumor agents [88]. Additionally, silver complexes may encounter issues such as low light stability and aqueous solubility [89]. Hence, careful selection of ligands is essential to prepare complexes with biologically compatible properties. It is noteworthy that certain silver complexes, particularly those with diphosphine and N-heterocyclic carbene ligands, have been reported to demonstrate promising in vivo antitumor activity in mice afflicted with leukemia, reticular cell sarcoma, and ovarian cancer [90].

A stable silver (I) polymeric complex with 1,8-dithia-4,11-diazacyclotetradecane has been reported, remaining stable under air, light, and in solution. This complex has been studied for its anti-breast CSC activity in both monolayer and three-dimensional cell culture models [91]. The silver compound demonstrated effectiveness against CSCs through multiple mechanisms. It penetrates the cytosol, increases intracellular ROS levels, and triggers caspase-dependent apoptosis, leading to CSC death. The compound was also internalized by breast CSCs, mainly found in the cytoplasm and cell membrane, suggesting that its targets are primarily cytoplasmic biomolecules rather than genomic DNA. Additionally, interaction studies revealed that the compound forms insoluble precipitates with thiol-containing biomolecules, such as proteins [39]. These combined mechanisms, including cellular entry, ROS elevation, and interactions with biomolecules, contribute to the efficacy of the silver compound in eradicating CSCs, offering novel insights into therapeutic strategies targeting these resistant cells.

#### 3.1.8. Osmium

These complexes operate through distinct mechanisms compared to platinum-based drugs, offering diverse cytotoxicity profiles and the ability to overcome both intrinsic and acquired resistance commonly observed in various tumors [92]. Although osmium’s reputation for high toxicity has limited its exploration as an anticancer agent [81], recent advancements have highlighted promising compounds. Half-sandwich “piano-stool” osmium (II) arene complexes have shown significant in vitro activity without encountering cisplatin cross-resistance [93]. Additionally, osmium (VI) nitride compounds with tridentate Schiff bases and monodentate azole heterocycle ligands have demonstrated potential in both in vitro and in vivo studies [29].

A half-sandwich complex [Os(η^6^-p-cym)(bphen)(dca)]PF_6_ (Os(II) complex) [p-cym = 1-methyl-4-(propan-2-yl)benzene (*p*-cymene), bphen = 4,7-diphenyl-1,10-phenanthroline (bathophenanthroline); dca = dichloroacetate] demonstrated remarkable activity against highly aggressive triple-negative breast cancer cells (MDA-MB-231) [94]. Consequently, this prompted an investigation into the in vitro efficacy of these Os (II) half-sandwich complexes against human breast CSCs (hBCSCs). The findings revealed that the mentioned Os (II) complex exhibits greater effectiveness against hBCSCs compared to conventional salinomycin, a well-established potent CSC agent recognized for its selective potency against CSCs [95,96].

Research on osmium (VI) nitride complexes identified a complex with notable potential, effectively reducing CSCs in breast cancer cell populations and inhibiting mammosphere formation, showing results similar to salinomycin, a known CSC-targeting agent [54]. Mechanistic studies revealed that the complex induces DNA damage and endoplasmic reticulum stress, which is key to its selectivity for CSCs. Further studies demonstrated that an Os (II) complex with a dca ligand also eradicated CSCs in MCF-7 and SKBR-3 human breast cancer cells, targeting CD44-positive, CD24-negative CSC-like cells and inhibiting mammosphere formation. The Os (II) complex was more effective than salinomycin in eliminating CSCs from these cell populations [48].

Encouragingly, the CSC-specific potency displayed by osmium (II) complexes poses a challenge to some of the most CSC-selective compounds identified to date. Additionally, they inhibit mammosphere formation by specifically targeting CD44-positive CSC-like cells. Considering these discoveries and the pressing medical necessity for CSC-specific chemotherapies to combat cancer relapse and metastasis formation in clinical settings, the anti-CSC properties of osmium (II) complexes hold significant preclinical promise for the development of new research endeavors [54].

#### 3.1.9. Ruthenium

Ruthenium complexes containing bipyridine and terpyridine ligands, along with an exchangeable (reactive) coordination site ([Ru(terpy)(bpy)X]n+), can interact with solvent-accessible guanines in DNA. Unlike other standard DNA metallating agents, such as cisplatin, these complexes exhibit kinetically controlled reactivity with DNA, likely due to the steric bulk provided by their ruthenium ligands, which also facilitates exceptional chemoselectivity. Furthermore, these ruthenium complexes have been shown to effectively bind solvent-exposed guanines at adjacent positions in four-stranded guanine–DNA quadruplexes. Detailed mechanistic studies with pancreatic tumor stem cells have demonstrated that ruthenium complexes can specifically target mitochondria and interact with their DNA [31,61].

Ru (II)–p-cymene complexes derived from mesalazine derivatives demonstrate regulatory effects on stemness gene expression in CSCs through unique properties and interactions. These complexes effectively inhibit the growth of colon CSC spheroids by targeting specific genes involved in stemness regulation. They have been observed to downregulate key stemness regulators, such as SOX2, KLF4, and Oct4 in CSCs, disrupting their self-renewal and survival mechanisms. Additionally, the complexes modulate the expression of efflux transporters such as ABCG2, which contribute to drug resistance in CSCs, potentially overcoming mechanisms that aid cancer cell survival during chemotherapy. Unlike oxaliplatin, these complexes do not enhance the expression of HES-1, a crucial gene in the Notch signaling pathway implicated in colorectal cancer self-renewal and tumorigenicity, suggesting they may disrupt the signaling mechanisms supporting cancer stemness [41]. Overall, the mechanism by which these complexes regulate the expression of stemness genes in CSCs involves inhibiting key regulators, modulating efflux transporter activity, and potentially interfering with signaling pathways that maintain stem cell properties.

#### 3.1.10. Gold

Gold complexes have demonstrated promising anticancer activity in several cancer cell lines, including lung cancer (A549), breast cancer (MCF-7), prostate cancer (PC-3), osteosarcoma (MG-63), and ovarian cancer (A2780 and A2780cis). Studies suggest that the cytotoxicity of gold(I)–carbene complexes, particularly those containing the IPr moiety, is associated with a more lipophilic nature and enhanced mitochondrial disruption. For example, cationic bis–carbene complexes, such as [Au(IPr)_2_]BF_4_, have been found to be more effective than their neutral counterparts, such as [Au(IPr)Cl], in inducing cancer cell death, mediated by apoptotic pathways. Furthermore, dinuclear gold(I) complexes based on carbene and diphosphane ligands, such as the bis [2-(dicyclohexylphosphane)ethyl] amine complex, have demonstrated potent cytotoxic activity against several cell lines. These complexes showed comparable antitumor efficacy against cisplatin-sensitive ovarian cancer cells (A2780) and against A2780cis cells, a cisplatin-resistant epithelial human ovarian cell line [32].

Gold complexes interact with redox control system proteins and/or the proteasome machinery, suggesting that their anticancer activity primarily stems from these two targets [97]. Specifically, gold (I) complexes can bind to the selenocysteine residue at the C-terminal active site of the enzyme thioredoxin reductase (TrxR), a well-known target for the cytotoxic effects of gold compounds [98]. TrxR acts as a scavenger of oxidants and free radicals, providing protection against oxidative stress damage. Additionally, another significant target for various gold complexes is the 20S proteasome. As the central component of the protein degradation machinery, known as the ubiquitin–proteasome system (UPS), its inhibition by gold (I) complexes can disrupt protein homeostasis and impair cell survival [99,100].

## 4. Discussion

- Antitumor Activity of Coordination Complexes Against Cancer Stem Cells: A Cytotoxic Perspective

The results presented in Table 3 reveal significant variations in the cytotoxic efficacy of the tested metal complexes, as evidenced by the IC_50_ values across different cancer cell lines and populations enriched in cancer stem cells. Platinum-based compounds, such as tri-platinum (II) and mononuclear complexes, exhibited low IC_50_ values in breast cancer cell lines, suggesting high potency, particularly against CSC-enriched mammospheres. This effect is likely related to these compounds’ ability to intercalate DNA and induce direct genotoxic stress, thereby triggering mitochondrial-dependent (intrinsic) apoptotic pathways.

In contrast, copper complexes, especially those coordinated with phenanthroline ligands and NSAID derivatives, showed more variable efficacy, highlighting that cytotoxic potential is strongly influenced by the chemical nature of the ligand and its capacity to generate reactive oxygen species, which contribute to apoptosis via oxidative stress and disruption of redox homeostasis. Additionally, the differential sensitivity observed among HMLER, HMLER-shEcad, and mammosphere cultures suggests that CSC-associated markers (e.g., CD44^+^/CD24^−^) and cellular context play key roles in modulating therapeutic response. Lower IC_50_ values in CSC-enriched models imply greater compound selectivity or enhanced vulnerability of CSCs to specific metal-based agents. These findings underscore the relevance of tumor phenotype and microenvironment in evaluating compound efficacy. Although molecular mechanisms require further elucidation, evidence from the literature indicates that metal complexes can activate multiple cell death pathways, including caspase cascade activation, mitochondrial dysfunction, and ROS-mediated apoptosis—mechanisms differentially modulated by the chemical structure of the complex and the biological features of the target cells.

Despite the promising cytotoxic profiles of metal-based complexes against CSCs, a comprehensive assessment of their safety remains critical. Metal coordination compounds, such as those based on copper, cobalt, platinum, and iridium, can exhibit substantial toxicity toward non-cancerous cells. For instance, the copper (II)–phenanthroline complexes evaluated by Vasconcelos et al. demonstrated remarkable potency against CSC-enriched HMLER-shEcad mammospheres (IC_50_ = 0.11 µM); yet, they showed comparable cytotoxicity in non-tumorigenic HEK293 cells (IC_50_ = 0.09 µM), indicating limited selectivity [34]. Similarly, the silver (I) polymeric complex analyzed was active against CSCs (IC_50_ = 12.95 µM) but also induced cytotoxicity in normal human bronchial epithelial cells (BEAS-2B, IC_50_ = 8.66 µM) and non-tumorigenic mammary cells (MCF-10A, IC_50_ = 10.12 µM) [37]. These findings raise concerns about the therapeutic window and cellular specificity of such compounds. Furthermore, the risks of bioaccumulation and systemic toxicity present significant challenges for clinical translation. For example, some copper and cobalt complexes displayed inconsistent selectivity across cell types, maintaining considerable toxicity toward normal cells despite modest anticancer effects [39]. The literature also reports adverse effects commonly associated with platinum and nickel-based agents, such as nephrotoxicity and neurotoxicity, often mediated by oxidative stress, mitochondrial dysfunction, or DNA adduct formation. Therefore, future studies should prioritize rigorous in vitro and in vivo toxicity evaluations and explore strategies to enhance tumor selectivity, including nanoparticle delivery systems, controlled-release formulations, or conjugation with ligands targeting CSC-specific markers, to minimize off-target effects and improve translational potential.

- Mechanisms of action of metal complexes leading to induced cell death

Recent studies have highlighted the therapeutic potential of metal complexes, particularly copper (II) complexes, in targeting cancer stem cells (CSCs). Complexes containing terpyridine and p-toluene sulfonamide ligands have demonstrated the ability to penetrate breast CSCs, accumulating in the cytoplasm and elevating intracellular reactive oxygen species (ROS) levels. This oxidative stress partially disrupts the endoplasmic reticulum, triggering apoptosis [33]. Similarly, binuclear copper (II)–phenanthroline complexes induce rapid ROS elevation, leading to oxidative DNA damage and caspase-dependent apoptotic pathways [34].

The overexpression of cyclooxygenase-2 (COX-2) in cancer cells contributes to tumor proliferation and resistance to conventional chemo- and radiotherapies. Its pivotal role in CSC biology, mediating tumor repopulation and metastasis, makes COX-2 an attractive molecular target for CSC-directed therapies [33]. In this context, copper (II)–NSAID complexes have been structurally optimized to maintain biological stability while effectively inducing apoptosis in breast CSCs through ROS generation and COX-2 inhibition [46,47]. The cytotoxicity is attributed to ROS production, possibly via Fenton-like reactions catalyzed by reduced copper species, while the released NSAID ligands inhibit COX-2 activity, reinforcing the dual mechanism of action. Moreover, tetranuclear copper (II) complexes have exhibited efficacy in simultaneously eliminating bulk tumor cells and CSCs, suggesting enhanced therapeutic scope [52]. This dual activity is further supported by cobalt (III)–polypyridyl complexes conjugated with diflunisal, which downregulate COX-2 expression and contribute to cytotoxic effects [49].

Beyond apoptosis, some metal complexes induce immunogenic cell death (ICD) in breast CSCs, enhancing their clearance by macrophages. For instance, binuclear copper (II)–NSAID complexes promote ICD markers, such as extracellular ATP and HMGB-1 release, while cobalt (III)–cyclam conjugates with flufenamic acid exhibit similar immunotherapeutic potential [30,55]. This immunogenic mechanism opens avenues for combinatorial strategies integrating metal complexes with immune modulation.

Nickel (II) complexes with polypyridyl ligands and NSAIDs also induce necroptosis, a programmed immunogenic cell death pathway linked to COX-2 signaling [35]. The reduction in cytotoxicity upon necroptosis inhibition underscores the pathway’s involvement in CSC death [42].

Platinum (II) and palladium (II) complexes efficiently penetrate mammospheres, damaging genomic DNA and activating caspase-dependent apoptosis, thereby targeting CSC populations [39]. Bioinspired macrocyclic copper (II) complexes similarly induce ROS, inhibit COX-2, and activate JNK and p38 pathways, culminating in apoptosis [37].

Notably, iridium (III) cyclometalated complexes selectively localize to mitochondria in glioma stem cells, inducing apoptosis via mitochondrial membrane depolarization, ROS elevation, and caspase activation [40,53].

Given the increasing resistance of CSCs to apoptosis, alternative cell death pathways have gained attention. A nickel (II)–dithiocarbamate–phenanthroline complex triggers necroptosis in breast CSCs via plasma membrane disruption and mitochondrial depolarization, independent of ROS or PARP-1 activity but dependent on RIP1-RIP3-MLKL necrosome assembly [5]. Likewise, osmium (II) complexes induce necroptosis, while ruthenium (II) complexes combined with salinomycin inhibit both necroptosis and apoptosis. This functional divergence may relate to osmium (II)’s capacity to transport biologically active dichloroacetic acid intracellularly [48].

Together, these findings illustrate the versatility of metal complexes as multifaceted agents capable of disrupting cancer stem cell (CSC) survival through various mechanisms, including induction of oxidative stress, DNA damage, caspase activation, COX-2 inhibition, immune modulation, necroptosis, and immunogenic cell death. The ability of these compounds, particularly those based on copper, nickel, cobalt, and noble metals, to simultaneously target multiple cellular pathways, such as reactive oxygen species (ROS) generation and activation of the JNK/p38 pathways, reinforces their potential as therapeutic agents directed at CSCs, which are responsible for tumor resistance and relapse. The integration of both apoptotic and non-apoptotic cell death pathways, combined with immunogenic effects, highlights the promising role of these complexes in the development of advanced and more effective CSC-targeted therapies.

However, despite significant in vitro advances, the translation of these findings into in vivo models remains limited, constraining the evaluation of systemic impact, bioavailability, and toxicity of these complexes. Furthermore, although selective targeting of CSCs has been suggested, few studies have thoroughly investigated the molecular mechanisms conferring this selectivity, highlighting the need for deeper research to minimize the side effects on normal cells. Another relevant aspect is the complexity of the involved cell death pathways, such as necroptosis and immunogenic cell death, which represent promising strategies to overcome apoptosis resistance.

Therefore, the integration of various pathways associated with cell death underscores the potential of metal complexes as promising strategies for the targeted treatment of tumor stem cells. The majority of analyzed studies have identified this interaction, presenting significant mechanistic insights that demonstrate interference in oxidative stress, which is closely linked to apoptosis, DNA fragmentation, activation of caspases, among other pathways.

- Modulation of tumor stem cell markers by the metallocomplex

The markers of tumor stem cells are crucial molecules or proteins that confer stem-cell-like properties to cancer cells, serving as essential tools for identification, characterization, and therapeutic targeting. Their expression profiles vary significantly according to the tumor type and progression stage, reflecting the inherent complexity and heterogeneity of CSC biology. Among the most frequently reported markers in the analyzed studies are Epcam, CD44, CD133, CD24, SOX2, KLF4, Oct4, CXCR4, NOTCH1, ALDH1, and HES1 [31,32,36,41,48,54].

Experimentally, CSC enrichment was predominantly achieved by selecting cells expressing specific surface markers. For instance, the HMLER breast cancer cell line, which naturally overexpresses CD44, was treated with low-dose paclitaxel (10 nM for four days), leading to a subpopulation enriched in CD44+ cells (>30%), termed HMLER fiscal cells [54]. This approach effectively mimics chemoresistance-driven CSC enrichment, emphasizing the plasticity of tumor cells under therapeutic pressure. Similarly, human rhabdomyosarcoma RD cells enriched for CD133+ expression demonstrated enhanced self-renewal capacity, as evidenced by increased single-cell spheroid formation compared to their CD133- counterparts [36]. This highlights the functional relevance of CD133 as a CSC marker associated with tumor propagation.

In colon cancer models, contrasting effects of conventional chemotherapeutics and metal-based complexes on CSC populations were reported. Oxaliplatin reduced spheroid size but paradoxically increased the expression of stemness-related genes and the ABCG2 transporter, suggesting an inadvertent enrichment of CSC-like traits and potential chemoresistance [41]. Conversely, salinomycin also diminished spheroid size but without elevating stemness markers, indicating selective cytotoxicity against CSCs. Notably, ruthenium complexes, such as [Ru(II)(p-cymene)X_2_]^2+^ (X = Cl, I), demonstrated potent inhibition of CSC growth without upregulating ABCG2 or stemness genes, underscoring their promise as more targeted anti-CSC agents.

Lung cancer studies further reveal the complex interplay between metal complexes and CSC markers. The treatment of A549 cells with a gold(I) complex reduced ALDH1 enzymatic activity and lowered CD133 expression, the key indicators of CSC presence and function. The same complex also decreased CD44 levels, impacting cell adhesion, growth, resistance to cisplatin, and metastatic potential [32,101]. However, an unexpected upregulation of the immunosuppressive molecule PD-L1 was observed, which may complicate therapeutic outcomes by facilitating immune evasion. Importantly, a dose-dependent reduction in NOTCH1 expression was noted, suggesting interference with a critical CSC regulatory pathway linked to chemotherapy resistance and poor prognosis.

In breast cancer, cisplatin treatment of MCF-7 cells slightly increased the CSC-enriched CD44+/CD24− subpopulation, indicating potential CSC enrichment after therapy. Ruthenium complexes [Ru(η6-pcym)(bphen)(dca)]PF6 showed limited efficacy in reducing this CSC fraction, whereas salinomycin and osmium complexes [Os(η^6^-p-cym)(bphen)(dca)]PF_6_ significantly decreased the CD44+/CD24− population, pointing to their superior potential for CSC targeting [48]. Complementary analyses of SKBR-3 cells revealed that osmium complexes were more effective than ruthenium in reducing ALDH-positive CSC-like cells, suggesting that subtle variations in metal center and ligand environment can critically influence CSC selectivity and therapeutic efficacy.

In vivo investigations provide encouraging, albeit preliminary, evidence of metal complexes’ additive or synergistic effects when combined with established chemotherapeutics. For example, ruthenium complexes combined with gemcitabine in pancreatic ductal adenocarcinoma (Panc185) models not only delayed tumor growth but also significantly reduced EpCAM+ epithelial tumor cells and the CD133+ and CD133+/CXCR4+ CSC subpopulations, reinforcing the translational relevance of these compounds [31]. Similar growth inhibition was observed in colorectal and osteosarcoma models, tumors known to be driven by CSC populations, further validating ruthenium complexes as promising agents for multi-targeted cancer therapies [102].

Taken together, these findings illustrate the multifaceted abilities of metal complexes, especially those based on ruthenium, osmium, gold, and other transition metals, to modulate key CSC markers and functions across various tumor types. However, the heterogeneous responses, such as paradoxical marker upregulation or immunosuppressive effects, highlight the need for comprehensive mechanistic studies to optimize their clinical utility and minimize unintended consequences. Moreover, the selective targeting of CSCs versus bulk tumor cells remains an ongoing challenge requiring further elucidation of underlying molecular pathways. Addressing these gaps will be critical for advancing metal complexes from promising in vitro tools to effective and safe CSC-directed therapies in clinical oncology.

- Limitations, Translational Challenges, and Future Research Opportunities

Although the 27 studies included in this review all focus on cancer stem cells, a key limitation lies in the relatively small number of investigations that specifically assess the effects of metal-based compounds directly on CSC populations. This reflects a broader gap in the literature and underscores the need for further research dedicated exclusively to this subpopulation, particularly through robust and targeted experimental models.

Despite this limitation, selected studies show promising in vitro results against CSCs. However, the clinical application of these metal-based compounds remains a challenge. One major barrier is the paucity of preclinical and clinical studies evaluating CSC-targeted agents, which hinders a comprehensive understanding of their efficacy and safety in vivo. Furthermore, the inherent complexity of CSC biology, including phenotypic plasticity, tumor heterogeneity, and resistance mechanisms, requires further investigation to guide the rational design of selective and effective therapies. Other obstacles include pharmacokinetic limitations, potential off-target toxicity, and regulatory challenges commonly associated with metal-based therapies.

Nonetheless, the findings compiled in this review identify relevant molecular targets and suggest innovative strategies that may inform future translational research. By enhancing our understanding of CSC-specific pathways, these compounds hold potential to contribute to the development of more effective and personalized anticancer therapies. Therefore, while acknowledging the current scarcity of highly focused studies, this review offers valuable insights and reinforces the importance of expanding research in this emerging and promising field.

Despite valuable insights, it is important to recognize that the current literature on metal complexes specifically tested in cancer stem cells remains limited. Most studies to date involve in vitro models with small sample sizes, and although the results are promising, the lack of broader, standardized protocols limits the generalizability and clinical applicability of the results. Therefore, future studies should aim to validate these mechanisms in more diverse CSC populations and in clinically relevant in vivo models. Furthermore, expanding the study of how metal complexes affect CSC-related pathways, such as immunogenic cell death, mitochondrial disruption, and necroptosis, may uncover novel therapeutic targets. Exploring the synergistic effects of metal complexes with standard therapies and immune modulators may also pave the way for combination strategies with greater translational potential. These directions may help overcome CSC-associated resistance and recurrence, ultimately enhancing treatment outcomes.

## 5. Conclusions

The studies examined in this systematic review provide substantial evidence that stem cell subpopulations within cancerous tumors are impacted by treatments involving metal coordination compounds, either synthesized alone or in combination with other molecules. Notably, nearly all reviewed studies used the in vitro formation of stem cells as a strategic approach, achieved through the use of nutrient-enriched culture media, which promotes the expression of stem cell markers in cancer-derived cells. Although there is a growing understanding of the factors influencing the response to coordination compound treatments in cells exhibiting stem-cell-like characteristics, it remains essential to emphasize the need for further research, particularly in vivo studies that replicate the complexity of biological processes. Additionally, a multifaceted approach is necessary to refine therapeutic strategies and improve clinical outcomes for patients with various cancer types.

In the general screening conducted to assess the cytotoxicity of various complexes, the most promising candidates were identified as follows: copper (II)–phenanthroline complexes demonstrated potent activity in breast tumor cells (HMLER-shEcad mammospheres); nickel (II)–flufenamic acid complexes, combined with 4,7-diphenyl-1,10-phenanthroline, showed significant effects in osteosarcoma tumor stem cells; the platinum complex (cis,trans,cis-[Pt(NH_3_)_2_(cinn)_2_Cl_2_] (2)) exhibited notable cytotoxicity in rhabdosarcoma tumor cells; Ru (II)–p-cymene complexes, featuring an imidazole-mesalazine Schiff base (II), were effective in colorectal cancer tumor stem cells; and dinuclear gold complexes (I) displayed strong cytotoxic potential in lung tumor stem cells.

Only two lines of research have advanced their investigations with the complexes in mouse models. In an immunocompetent 4T1 mouse model of metastatic triple-negative breast cancer, treatment with a cobalt (III)–cyclam complex conjugated to two flufenamic acid moieties demonstrated promising in vivo effects. The treated group exhibited a significant reduction in lung tumor colonization compared to the control group, suggesting that the complex effectively prevents the metastasis of breast cancer cells to the lungs. No significant differences in organ cellularity, including the heart, liver, spleen, or kidneys, were observed between the treated and control groups, indicating a favorable safety profile. Overall, the results show that the cobalt (III)–cyclam complex with flufenamic acid is effective in reducing tumor growth and inhibiting lung metastasis without inducing significant systemic toxicity [31].

Additionally, the ruthenium complex [Ru(terpy)(bpy)(H_2_O)]Cl_2_ was evaluated in a syngeneic in vivo model of pancreatic ductal adenocarcinoma, demonstrating promising tumor growth inhibition by disrupting ATP generation. However, its exact mechanism of action remains unclear, although it is likely not mediated through the inhibition of CSCs. Preliminary experiments indicate that both complexes show effective in vivo activity, highlighting their significant preclinical potential.

## Figures and Tables

**Table 1:** Articles selected for the systematic review following the application of the PRISMA methodology.

Selected Articles	Year
1. A Breast Cancer Stem Cell-Selective, Mammospheres-Potent Osmium (VI) Nitrido Complex	2014
2. The Breast Cancer Stem Cell Potency of Copper (II) Complexes Bearing Nonsteroidal Anti-Inflammatory Drugs and Their Encapsulation Using Polymeric Nanoparticles	2016
3. Cancer Stem Cell and Bulk Cancer Cell Active Copper (II) Complexes with Vanillin Schiff Base Derivatives and Naproxen	2017
4. A Reactive Oxygen Species-Generating, Cyclooxygenase-2 Inhibiting, Cancer Stem Cell-Potent Tetranuclear Copper (II) Cluster	2017
5. Induction of Necroptosis in Cancer Stem Cells Using a Nickel (II)-Dithiocarbamate Phenanthroline Complex	2017
6. Highly Charged, Cytotoxic, Cyclometalated Iridium (III) Complexes as Cancer Stem Cell Mitochondriotropics	2018
7. Diflunisal-Adjoined Cobalt (III)-Polypyridyl Complexes as Anti-Cancer Stem Cell Agents	2018
8. Modulating the Chemical and Biological Properties of Cancer Stem Cell-Potent Copper (II)-Nonsteroidal Anti-Inflammatory Drug Complexes	2019
9. An Anticancer Os (II) Bathophenanthroline Complex as a Human Breast Cancer Stem Cell-Selective, Mammosphere Potent Agent That Kills Cells by Necroptosis	2019
10. A Triangular Platinum (II) Multinuclear Complex with Cytotoxicity Towards Breast Cancer Stem Cells	2019
11. Platinum (IV) Derivatives with Cinnamate Axial Ligands as Potent Agents Against Both Differentiated and Tumorigenic Cancer Stem Rhabdomyosarcoma Cells	2020
12. Breast Cancer Stem Cell Potency of Nickel (II)-Polypyridyl Complexes Containing Non-Steroidal Anti-Inflammatory Drugs	2020
13. Identification of Two Mitochondrial-Targeting Cyclometalated Iridium (III) Complexes as Potent Anti-Glioma Stem Cell Agents	2020
14. The Discrete Breast Cancer Stem Cell Mammosphere Activity of Group 10-Bis (Azadiphosphine) Metal Complexes	2020
15. A Tri-Metallic Palladium Complex with Breast Cancer Stem Cell Potency	2020
16. Inhibition of 3D Colon Cancer Stem Cell Spheroids by Cytotoxic Ru(II)-p-Cymene Complexes of Mesalazine Derivatives	2020
17. Combination of Histone Deacetylase Inhibitor with Cu (II) 5,5-Diethylbarbiturate Complex Induces Apoptosis in Breast Cancer Stem Cells: A Promising Novel Approach	2021
18. A Dithiacyclam-Coordinated Silver (I) Polymer with Anti-Cancer Stem Cell Activity	2021
19. An Osteosarcoma Stem Cell-Potent Nickel (II)-Polypyridyl Complex Containing Flufenamic Acid	2022
20. Dipyridophenazine Iridium (III) Complex as a Phototoxic Cancer Stem Cell Selective, Mitochondria Targeting Agent	2022
21. A Bioinspired Redox-Modulating Copper (II)-Macrocyclic Complex Bearing Non-Steroidal Anti-Inflammatory Drugs with Anti-Cancer Stem Cell Activity	2022
22. The Bulk Breast Cancer Cell and Breast Cancer Stem Cell Activity of Binuclear Copper (II)-Phenanthroline Complexes	2023
23. A Breast Cancer Stem Active Cobalt (III)-Cyclam Complex Containing Flufenamic Acid with Immunogenic Potential	2023
24. Dinuclear Gold (I) Complexes Based on Carbene and Diphosphane Ligands: Bis [2-(Dicyclohexylphosphano)ethyl]amine Complex Inhibits Proteasome Activity, Decreases Stem Cell Markers and Spheroid Viability in Lung Cancer Cells	2023
25. Targeting Cancer Stem Cell OXPHOS with Tailored Ruthenium Complexes as a New Anti-Cancer Strategy	2024
26. An Immunogenic Anti-Cancer Stem Cell Bi-Nuclear Copper (II)-Flufenamic Acid Complex	2024
27. Cobalt (III)-Macrocyclic Scaffolds with Anti-Cancer Stem Cell Activity	2024

**Table 2:** Summary of characteristics of the included studies.

First Author (Year)	Country	Cancer Type	CSC Markers	Coordination Complex ^1^	Type of Cell Death ^2^
Fang, 2024 [28]	UK	Breast cancer	–	[Co(1,4,7,11-tetraazacyclotetradecane)Cl2]+ [Co(1-oxa-4,8,12-triazacyclotetradecane)Cl2]+	Induces cell death (unspecified)
Fang, 2024 [29]	UK	Breast cancer	–	[Co(cyclam)Cl_2_]^+^, [Co(oxacyclam)Cl_2_]^+^	Apoptosis
Li, 2024 [30]	UK	Breast cancer	–	Cu (II)–flufenamic acid complexes	Induces cell death (unspecified)
Alcalá, 2024 [31]	Spain	Pancreatic, colorectal, osteosarcoma	CD133, EpCAM, CXCR4	Ru (II) complexes	Induces cell death (unspecified)
Casagrande, 2023 [32]	Italy	Lung cancer	NOTCH1, CD133, ALDH1, CD44	Dinuclear Au(I) complex	Apoptosis
Singh, 2023 [33]	UK	Breast cancer	–	Cu (II)–terpyridine complexes	Apoptosis
Osei, 2023 [34]	UK	Breast cancer	C117	Cu (II)–phenanthroline complexes	Apoptosis
Passeri, 2022 [35]	UK	Osteosarcoma	C117	Ni (II)–flufenamic acid + phenanthroline complexes	Necroptosis
Markova, 2022 [36]	Czech Republic	Rhabdomyosarcoma	CD133	Ir (III)–benzimidazole complex	Necrosis
Johnson, 2022 [37]	UK	Breast cancer	–	Cu (II)–cyclam + NSAID complexes	Induces cell death (unspecified)
Erkisa, 2021 [38]	Turkey	Breast cancer	–	Cu (II)–valproic acid–barbital complex	Apoptosis
Johnson, 2021 [39]	UK	Breast cancer	–	Polymeric Ag(I) complex	Apoptosis
Peng, 2020 [40]	China	Glioblastoma	–	Cyclometalated Ir(III) complexes	Apoptosis
Acharya, 2020 [41]	India	Colon cancer	SOX2, KLF4, Oct4, HES1	[Ru(p-cymene)X_2_]_2_ (X = Cl, I)	Induces cell death (unspecified)
Feld, 2020 [42]	UK	Breast cancer	–	Ni (II)–NSAID complexes	Necroptosis
Xiao, 2020 [43]	UK	Breast cancer	–	Pt(II), Pd(II), Ni(II) complexes	Apoptosis
Zajac, 2020 [44]	Czech Republic	Rhabdomyosarcoma	CD44, CD133	Pt(NH_3_)_2_(OH)(cinn)Cl_2_ complexes	Induces cell death (unspecified)
Eskandari, 2020 [45]	UK	Breast cancer	–	Trimetallic Pd complex	Apoptosis
Shin, 2019 [46]	UK	Breast cancer	–	Cu–NSAID complex	Apoptosis
Zhang, 2019 [47]	UK	Breast cancer	-	Copper (II) coordination complexes containing NSAIDs	Apoptosis
Novohradsky, 2019 [48]	Czech Republic	Breast cancer	C44 and CD24	Os (II)/Ru (II)–p-cymene–bphen–dca complex	Induces cell death (unspecified)
Abe, 2018 [49]	UK	Breast cancer	-	Cobalt (III)–diflunisal–polypyridyl complex	Apoptosis
Laws, 2018 [50]	UK	Breast cancer	-	Iridium (III)–polypyridyl–pyridinium complex	Apoptosis
Lu, 2017 [51]	UK	Breast cancer	-	Copper (II)–vanillin–naproxen Schiff base complex	Induces cell death (unspecified)
Lu, 2017 [52]	UK	Breast cancer	-	Tetranuclear copper (II) complex	Induces cell death (unspecified)
Eskandari, 2016 [53]	UK	Breast cancer	-	Copper (II)–phenanthroline–NSAID complex	Induces cell death (unspecified)
Suntharalingam, 2014 [54]	USA	Breast cancer	CD44	Osmium (VI) Nitrido complex	Apoptosis

^1^ Coordination complex: Only simplified names are presented here; detailed formulae and structures are available in the Supplementary Materials. ^2^ Type of cell death: If not specified in the original study, classified as “Induces cell death (unspecified)”.

**Table 3:** Values of IC_50_/μM tested on cancer lines and stem cells.

Studies Examining Breast Cancer								
**First author**	**Complex**		**HMLER-shEcad mammospheres** ^2^					
Xiao, 2020 [43]	Nickel (II) complex Palladium (II) complex Platinum (II) complex		2.98 ± 0.03 0.97 ± 0.09 0.24 ± 0.02					
**First author**	**Complex**		**HMLER**			**HMLER-shEcad ^1^**		
Suntharalingam, 2014 [54]	Osmium (VI) Nitrido complex (1) Osmium (VI) Nitrido complex (2) Osmium (VI) Nitrido complex (3)		11.20 ± 0.48 14.58 ± 0.20 82.80 ± 18.43			4.91 ± 0.86 16.06 ± 4.12 53.99 ± 2.45		
Lu, 2017 [51]	Copper (II) complexes with vanillin Schiff base derivatives and NSAIDs:		37.6 ± 3.3			36.0 ± 4.6		
Lu, 2017 [52]	Tetranuclear copper (II) complexes:		8.3 ± 0.5			8.4 ± 0.1		
Eskandari, 2016 [53]	Cu (bathocuproinedisulfonic acid disodium) (indomethacin)_2_ complex:		0.31 ± 0.01			0.22 ± 0.01		
**First author**	**Complex**		**HMLER**			**HMLER-shEcad ^1^**	**HMLER-shEcad mammospheres ^2^**	
Abe, 2018 [49]	Diflunisal–adjoined cobalt (III) polypyridyl complexes		3.9 ± 0.2			2.1 ± 0.1	22.8 ± 2.7	
	Mono-platinum complex		5.01 ± 0.03			7.01 ± 0.06	14.50 ± 0.91	
	Dinuclear platinum complex		2.59 ± 0.09			2.35 ± 0.01	16.00 ± 0.56	
	Tri-platinum (II) complex		2.24 ± 0.01			1.26 ± 0.03	4.55 ± 0.02	
Feld, 2020 [42]	Nickel (II)-3,4,7,8-tetramethyl-1,10-phenanthroline complexes bearing NSAID		2.74 ± 0.06			1.83 ± 0.11	55.40 ± 0.42	
Fang, 2024 [28]	[Co(1,4,7,11-tetraazacyclotetradecane)Cl2]+		4,64 ± 0.25			1,83 ± 0.32	51.46 ± 1.49	
	[Co(1-oxa-4,8,12-triazacyclotetradecane)Cl2]+		13,86 ± 0.01			3.09 ± 0.01	55.04 ± 3.23	
Eskandari, 2020 [45]	Tri-metallic palladium		2.24 ± 0.01			1.26 ± 0.03	4.55 ± 0.02	
Singh, 2023 [33]	Copper (ii) complexes with aryl-sulfonamide-functionalized terpyridine ligands		0.85 ± 0.04			0.69 ± 0.12	3.44 ± 0.03	
	Cobalt (III): [Co(1,4,7,11-tetraazaciclotetradecano)Cl_2_]^+^		4.64 ± 0.25			1.83 ± 0.32	51.46 ± 1.49	
	Cobalt (III): [Co(1-oxa-4,8,12 triazaciclotetradecano)Cl_2_]^+^		13.86 ± 0.01			3.09 ± 0.01	55.04 ± 3.23	
Li, 2024 [30]	Binuclear copper (II) complex comprising two copper (II) centers bound to flufenamic acid		3.57 ± 0.03			3.04 ± 0.09	9.17 ± 2.47	
	Binuclear Cu(II) complex with two Cu(II) centers coordinated to 3,4,7,8-tetramethyl-1,10-phenanthroline		0.31 ± 0.01			0.38 ± 0.17	0.74 ± 0.11	
Fang, 2024 [55]	Cobalt (III)–cyclam complex attached to two flufenamic acid moieties		0.27 ± 0.03			0.18 ± 0.003	0.27 ± 0.02	
	*Trans*-dichloro (cyclam)-cobalt(III) chloride		>100			>100	>133	
**First author**	**Complex**		**HMLER**	**HMLER-shEcad ^1^**		**HMLER-shEcad mammospheres ^2^**	**MDA-MB-231**	
Passeri, 2022 [35]	Nickel (II)–dithiocarbamate phenanthroline complexes		4.7 ± 0.2	4.7 ± 0.2		2.3 ± 0.2	3.3 ± 1.2	
**First author**	**Complex**		**HMLER**	**HMLER-shEcad ^1^**	**HMLER-shEcad mammospheres ^2^**	**U2OS**	**HepG2**	
Laws, 2018 [50]	Iridium (III) complexes bearing polypridyl and charged 1-methyl-2-(2-pyridyl) pyridinium ligands		5.4 ± 0.3	5.2 ± 0.1	21.0 ± 0.2	18.5 ± 3.0	65.4 ± 4.8	
**First author**	**Complex**		**MCF-7**	** MCF-7 CD44+/CD24− ^15^**		** SKBR-3 **	** SKBR-3 CD44+/CD24− ^16^**	
Novohradsky, 2019 [48]	Os (II) complex [Os(p-cym)(bphen)(dca)]PF_6_ and its Ru analog		2.6 ± 0.4	0.58 ± 0.07		1.0 ± 0.2	0.32 ± 0.09	
**First author**	**Complex**		**HMLER**	**HMLER-shEcad ^1^**		**HMLER-shEcad mammospheres ^2^**	**HEK 293**	
Osei, 2023 [34]	Copper (II)–phenanthroline complexes		0.03 ± 0.001	0.08 ± 0.004		0.11 ± 0.001	0.09 ± 0.003	
First author	**Complex**		**HMLER**	**HMLER-shEcad ^1^**	**HMLER-shEcad mammospheres ^2^**	**BEAS-2B**	**MCF10A**	**HEK 293**
Johnson, 2021 [39]	Silver (I) polymeric complex		4.58 ± 0.14	4.02 ± 0.35	12.95 ± 1.35	8.66 ± 0.48	10.12 ± 0.74	34.31 ± 0.10
Johnson, 2022 [37]	Copper (ii)–dithiacyclam and copper (ii)–cyclam complexes bearing NSAIDs		20.7 ± 0.2	12.5 ± 0.4	Nt* ^17^	57.1 ± 0.9	92.2 ± 1.2	57.2 ± 1.0
**Studies Examining Osteosarcoma**								
**First author**	**Complex**			** U2OS **		** U2OS-MTX ** **^3^**	** OSC-osteosphere ** **^4^**	
Passeri, 2022 [35]	Nickel (II)–flufenamic acid complexes with 4,7-diphenyl-1,10-phenanthroline			25.16 ± 0.40		26.90 ± 0.71	2.97 ± 0.04	
**Studies Examining Rhabdomyosarcoma**								
**First author**	**Complex**			**MCF-7 CD44− ^5^**	**MCF-7 CD44+ ^6^**	**RD CD133− ^7^**	**RD CD133+ ^8^**	
Zajac, 2020 [44]	*cis*,*trans*,*cis*-[Pt(NH_3_)_2_(OH)(cinn)Cl_2_] (1) *cis*,*trans*,*cis*-[Pt(NH_3_)_2_(cinn)_2_Cl_2_] (2)			12 ± 2 0.6 ± 0.1	17 ± 1 0.8 ± 0.1	8.4 ± 0.1 0.22 ± 0.04	12.0 ± 0.7 0.31 ± 0.06	
First author	**Complex**			** MRC-5 ** **^9^**		** DR ** **^10^**		
Markova, 2022 [36]	Ir (III) compound photoactivated by visible light of the type [Ir(C^N)_2_(dppz)][PF_6_] where C^N = 1-methyl-2-(2′-thienyl)benzimidazole			0.8 ± 0.1		0.046 ± 0.002		
**Studies Examining Glioblastoma Multiforme**								
**First author**	**Complex**	** GSC-3# ** **^11^**	** GSC-12# ** **^12^**	** GSC-18# ** **^13^**	** U87 **	** U251 **	** HAC **	** 293T **
Peng, 2020 [40]	Ir (III) (1) Ir (III) (2)	9.05 ± 0.9 7.55 ± 0.8	5.26 ± 0.5 5.40 ± 0.5	7.14 ± 0.7 8.80 ± 0.78	20.58 ± 2.1 31.04 ± 3.2	20.66 ± 2.2 27.15 ± 2.8	29.44 ± 3.0 27.87 ± 2.5	28.71 ± 2.8 26.25 ± 2.6
**Studies Examining Colorectal Cancer**								
**First author**	**Complex**			** HT-29 **	** MIAPaCa-2 **	** HepG2 **	** MDA-MB-231 **	
Acharya, 2020 [41]	Ru (II)–p-cymene complex of an imidazole-mesalazine Schiff base (I) Ru (II)–p-cymene complex of an imidazole-mesalazine Schiff base (II)			3.2 ± 0.3 2.6 ± 0.3	2.8 ± 0.1 2.3 ± 0.2	2.9 ± 0.3 2.4 ± 0.4	2.9 ± 0.4 2.2 ± 0.2	
**Studies Examining Lung Cancer**								
**First author**	**Complex**			** A549 **		** A549-MCTSs ^14^**		
Casagrande, 2023 [32]	Dinuclear gold(I) carbene complexes with IPr and diphosphane ligands (Dppe, Dppp, DCyPA)			0.13 ± 0.05		0.95 µM		

^1^ HMLER-shEcad: Human mammary epithelial cells with E-cadherin silencing, used to model cancer-stem-cell-like phenotypes. ^2^ HMLER-shEcad mammospheres: Spheroid cultures derived from HMLER-shEcad cells, enriched in CSCs due to anchorage-independent growth. ^3^ U2OS-MTX: Methotrexate-resistant U2OS subline, used to assess drug resistance. ^4^ OSC-osteosphere: Ovarian cancer stem-like spheroids, typically enriched in CSC characteristics. ^5^ MCF-7 CD44^−^: CD44-negative subpopulation of the MCF-7 breast cancer cell line. ^6^ MCF-7 CD44^+^: CD44-positive MCF-7 subpopulation with increased stemness and tumorigenic potential. ^7^ RD CD133^−^: CD133-negative RD rhabdomyosarcoma cells. ^8^ RD CD133^+^: CD133-positive subpopulation of RD cells, used to identify CSC traits. ^9^ MRC-5: Non-tumorigenic human lung fibroblast cell line used as a control for toxicity. ^10^ *DR:* Drug-resistant phenotype of unspecified cancer cell lines used to assess chemoresistance. ^11^ GSC-3#: Glioblastoma stem-like cell line 3, patient-derived and enriched for CSC markers. ^12^ GSC-12#: Glioblastoma stem-like cell line 12, used in studies on therapy resistance. ^13^ GSC-18#: Glioblastoma stem-like cell line 18, relevant for CSC-targeted research. ^14^ A549-MCTSs: Multicellular tumor spheroids derived from A549 lung carcinoma cells, mimicking 3D tumor architecture. ^15^ MCF-7 CD44^+^/CD24^−^: Subpopulation of MCF-7 breast cancer cells expressing CD44 and lacking CD24, commonly used as a CSC-enriched model. ^16^ SKBR-3 CD44^+^/CD24^−^: Subpopulation of SKBR-3 breast cancer cells with CD44 expression and CD24 depletion, indicative of CSC-like properties. ^17^ Nt*: Not tested. Note: The molecular structures of the complexes—Osmium(VI) nitride complexes (1–3), Ir(III) (1), and Ir(III) (2)—were not specified in the original article analyzed. These compounds are identified numerically for the purposes of this study.

## Supplementary Materials

The following supporting information can be downloaded at: https://www.mdpi.com/article/10.3390/pharmaceutics17070931/s1, Table S1: Descriptors used in the search strategy, Table S2: Critical analysis of preclinical studies, Table S3: Methodologies used in research, Graph S1: Quality analysis of all articles selected for systematic review, grouped by percentage of responses on the Likert scale.
