# Supplementary material for: Preclinical Trials of Cancer Stem Cells Targeted by Metal-Based Coordination Complexes: A Systematic Review
Source: Pharmaceutics. 2025 Jul 18;17(7):931. doi: 10.3390/pharmaceutics17070931 (PMC12299093; doi:10.3390/pharmaceutics17070931)
Supplement: Supplementary file 1 [file pharmaceutics-17-00931-s001.zip › pharmaceutics-3633521-supplementary.pdf]

Supplementary Material

Table S1 : Descriptors used in the search strategy.

Terms	PubMed (n) Papers	Nature (n) papers	Scielo (n) parpes	Google Scholar (n) parpes
**Databases from December 11 to 12, 2023**				
"Cancer Stem Cell"	11.925	4512	80	387.00
Coordenation Complex	102.322	1	*NF**	6.320.000
Metallic Coordination Compounds	20.814	1556	*NF**	669.000
**Keyword crossing**				
"Cancer Stem Cell" AND coordenation complex OR "Metallic Coordination Compounds"	20.814	2575	80	0
"Cancer Stem Cell" AND Complex	1.161	*NF**	*NF**	116.000
"Cancer Stem Cell" AND coordenation complex OR Metallocomplexes	90	*NF**	*NF**	7.780
"Cancer Stem Cell" AND Coordination Complex	61	119	*NF**	20.500
**Database Update January 27, 2025**				
"Cancer Stem Cell"	12.237	4468	163	448.000
Coordenation Complex	110.295	1	*NF**	5.060.000
Metallic Coordination Compounds	22.126	1841	*NF**	570.000
**Keyword crossing**				
"Cancer Stem Cell" AND coordenation complex OR "Metallic Coordination Compounds"	22.126	*NF**	163	0
"Cancer Stem Cell" AND Complex	1.264	2835	*NF**	119.000
"Cancer Stem Cell" AND coordenation complex OR Metallocomplexes	93	*NF**	*NF**	7.890
"Cancer Stem Cell" AND Coordination Complex	78	136	*NF**	7.890

NF*: Not Found

- **Assessment of the quality of articles : Brasil Ministério da Saúde, Secretaria de Vigilância em Saúde, Departamento de Saúde Ambiental, do Trabalhador e Vigilância das Emergências em Saúde Pública. *Training of Tutors in Field Epidemiology: Module 03—Introduction to Scientific Communication*. Ministry of Health: Brasília, Brazil, 2022.**

Critical analysis of preclinical studies

1 - The research question is original

a. I totally agree

b. I agree
c. Impartial
d. I disagree
e. I totally disagree

2 – The research works with ethical principles

a. I totally agree
b. I agree
c. Impartial
d. I disagree
e. I totally disagree

3 - The experimental model used was the most

a. I totally agree
b. I agree
c. Impartial
d. I disagree
e. I totally disagree

4- The method was clearly

a. I totally agree
b. I agree
c. Impartial
d. I disagree
e. I totally disagree

5 - The results were appropriately analyzed and described

a. I totally agree
b. I agree
c. Impartial
d. I disagree
e. I totally disagree

6 - The conclusions presented corroborate the answer to the research question

a. I totally agree
b. I agree
c. Impartial
d. I disagree
e. I totally disagree

Question 1, you should evaluate whether the research question is novel and relevant to contribute to scientific knowledge, in addition to being plausible and ethical. It should be supported by a coherent and logical theoretical framework to justify the study's development.

Question 2, this involves the principle of the 3 Rs by Russell and Burch (Reduce, Replace, and Refine), which propose reducing the number of animal experiments, minimizing the number of animals used, improving statistical treatment quality, replacing animal use with alternative methods, and refining techniques to reduce animal pain and suffering, including analgesia and asepsis care in procedures. Reflecting on the necessity of using these models is crucial given technological advancements and the availability of various alternative models.

Question 3, you should report all relevant study information such as species, strain, groups (control and experimental), baseline characteristics (body mass, sex, age, cell quantity), culture media, diet, doses,

solutions, reagents (purity, manufacturer), treatment duration (acute/chronic), and any other important information to ensure experiment reproducibility.

Question 4, you should evaluate whether the methodology description demonstrates that the chosen model effectively generates results that confirm the study hypotheses. The model choice should be accurate and justified.

Question 5, in vivo models must have a sufficient sample size for all groups, considering possible losses, which should be reported when they occur (investigation of specimen loss). Baseline differences, such as body mass between groups, should be considered in statistical analysis of models when relevant. In vitro assays should report cell culture contamination and cell viability, and experiments should be conducted in triplicate. These aspects guide methodological quality in studies and contribute to presenting reliable results.

Question 6, the conclusion must reference the research objectives, regardless of the methodology used.

Table S2: Critical analysis of preclinical studies

**Critical analysis of preclinical studies**							
		Items to be analyzed on the Likert scale					
Parpes	**Items**	**1**	**2**	**3**	**4**	**5**	**6**
**A Breast Cancer Stem Cell-Selective, Mammospheres-Potent Osmium (VI) Nitrido Complex**	a. I totally agree						
	b. I agree	100%	100%	67%	100%	100%	100%
	c. Impartial			33%			
	d. I disagree						
	e. I totally disagree						
**The breast cancer stem cell potency of copper (ii) complexes bearing nonsteroidal anti-inflammatory drugs and their encapsulation using polymeric nanoparticles**	a. I totally agree	100%					
	b. I agree		100%	100%	100%	100%	100%
	c. Impartial						
	d. I disagree						
	e. I totally disagree						
**Cancer Stem Cell and Bulk Cancer Cell Active Copper(II) Complexes with Vanillin Schiff Base Derivatives and Naproxen.**	a. I totally agree	100%	33%	100%		100%	
	b. I agree	33%	67%		67%		100%
	c. Impartial				33%		
	d. I disagree						
	e. I totally disagree						
**A reactive oxygen species-generating, cyclooxygenase-2 inhibiting, cancer stem cell-potent tetranuclear copper(ii) cluster**	a. I totally agree	33%					
	b. I agree	67%	100%	100%	66%		
	c. Impartial				33%	100%	100%
	d. I disagree						
	e. I totally disagree						
**Induction of Necroptosis in Cancer Stem Cells using a Nickel (II)-Dithiocarbamate Phenanthroline Complex**	a. I totally agree	100%		100%		100%	
	b. I agree		100%		33%		100%
	c. Impartial				67%		
	d. I disagree						
	e. I totally disagree						
**Highly Charged, Cytotoxic, Cyclometalated Iridium (III) Complexes as Cancer Stem Cell Mitochondriotropics**	a. I totally agree		100%				
	b. I agree	100%		100%		100%	100%
	c. Impartial				100%		
	d. I disagree						
	e. I totally disagree						
	a. I totally agree	33%					

**Diflunisal-adjoined cobal t(iii)-polypyridyl complexes as anti-cancer stem cell agentes**	b. I agree	67%	100%		100%	100%	
	c. Impartial						100%
	d. I disagree			100%			
	e. I totally disagree						
**Modulating the Chemical and Biological Properties of Cancer Stem Cell-Potent Copper (II)-Nonsteroidal Anti-Inflammatory Drug Complexes.**	a. I totally agree	100%		100%			
	b. I agree		100%				
	c. Impartial				33%	67%	100%
	d. I disagree				67%	33%	
	e. I totally disagree						
**An anticancer Os (II) bathophenanthroline complex as a human breast cancer stem cell-selective, mammosphere potent agent that kills cells by necroptosis**	a. I totally agree	100%					
	b. I agree		100%	100%	100%	100%	100%
	c. Impartial						
	d. I disagree						
	e. I totally disagree						
**Platinum (IV) Derivatives with Cinnamate Axial Ligands as Potent Agents Against Both Differentiated and Tumorigenic Cancer Stem Rhabdomyosarcoma Cells**	a. I totally agree	100%					
	b. I agree		100%	67%	100%	100%	100%
	c. Impartial			33%			
	d. I disagree						
	e. I totally disagree						
**A Triangular Platinum (II) Multinuclear Complex with Cytotoxicity Towards Breast Cancer Stem Cells**	a. I totally agree	100%				100%	
	b. I agree		100%				100%
	c. Impartial				100%		
	d. I disagree			100%			
	e. I totally disagree						
**Breast Cancer Stem Cell Potency of Nickel (II)-Polypyridyl Complexes Containing Non-steroidal Anti-inflammatory Drugs.**	a. I totally agree						
	b. I agree	100%	100%	100%		100%	100%
	c. Impartial				100%		
	d. I disagree						
	e. I totally disagree						
**Identification of two mitochondrial-targeting cyclometalated iridium(III) complexes as potent anti-glioma stem cells agents**	a. I totally agree	100%					
	b. I agree		100%	100%	100%	100%	100%
	c. Impartial						
	d. I disagree						
	e. I totally disagree						
**The Discrete Breast Cancer Stem Cell Mammosphere Activity of Group 10-Bis (azadiphosphine) Metal Complexes**	a. I totally agree						
	b. I agree	100%	100%	100%	67%	100%	
	c. Impartial				33%		100%
	d. I disagree						
	e. I totally disagree						
**A tri-metallic palladium complex with breast cancer stem cell potency.**	a. I totally agree						
	b. I agree	100%			100%	67%	
	c. Impartial		100%	100%		33%	
	d. I disagree						100%
	e. I totally disagree						
	a. I totally agree	100%					

**Inhibition of 3D colon cancer stem cell spheroids by cytotoxic RuII-p-cymene complexes of mesalazine derivatives**	b. I agree		100%	100%	100%	100%	100%
	c. Impartial						
	d. I disagree						
	e. I totally disagree						
**Combination of Histone Deacetylase Inhibitor with Cu (II) 5,5-diethylbarbiturate Complex Induces Apoptosis in Breast Cancer Stem Cells: A Promising Novel Approach.**	a. I totally agree	100%					
	b. I agree		100%	100%	100%	100%	67%
	c. Impartial						33%
	d. I disagree						
	e. I totally disagree						
**A dithiacyclam-coordinated silver (i) polymer with anti-cancer stem cell activity**	a. I totally agree						
	b. I agree	100%	67%	100%	100%	100%	100%
	c. Impartial		33%				
	d. I disagree						
	e. I totally disagree						
**An Osteosarcoma Stem Cell Potent Nickel (II)-Polypyridyl Complex Containing Flufenamic Acid**	a. I totally agree	100%					
	b. I agree		100%	100%	100%	100%	100%
	c. Impartial						
	d. I disagree						
	e. I totally disagree						
**Dipyridophenazine iridium (III) complex as a phototoxic cancer stem cell selective, mitochondria targeting agent**	a. I totally agree	100%				33%	
	b. I agree		100%	100%	67%	67%	100%
	c. Impartial				33%		
	d. I disagree						
	e. I totally disagree						
**A bioinspired redox-modulating copper (II)-macrocyclic complex bearing non-steroidal anti-inflammatory drugs with anti-cancer stem cell activity**	a. I totally agree	100%				100%	
	b. I agree		100%				100%
	c. Impartial			33%	100%		
	d. I disagree			67%			
	e. I totally disagree						
**Cancer stem cell activity of copper (II)-terpyridine complexes with aryl sulfonamide groups**	a. I totally agree						
	b. I agree	100%	100%	67%	100%	100%	
	c. Impartial			33%			100%
	d. I disagree						
	e. I totally disagree						
**The Bulk Breast Cancer Cell and Breast Cancer Stem Cell Activity of Binuclear Copper (II)-Phenanthroline Complexes**	a. I totally agree				100%		
	b. I agree	100%	100%			100%	67%
	c. Impartial			100%			33%
	d. I disagree						
	e. I totally disagree						
**Dinuclear gold(I) complexes based on carbene and diphosphane ligands: bis[2-(dicyclohexylphosphano)ethyl]amine complex inhibits the proteasome activity, decreases stem cell markers and spheroid viability in lung cancer cells.**	a. I totally agree	100 %	100 %	100 %	100 %	100 %	100 %
	b. I agree						
	c. Impartial						
	d. I disagree						
	e. I totally disagree						

**An immunogenic anti-cancer stem cell bi-nuclear copper(II)-flufenamic acid complex**	a. I totally agree	100%				33%	
	b. I agree		100%	100%	67%	67%	100%
	c. Impartial				33%		
	d. I disagree	100%				33%	
	e. I totally disagree		100%	100%	67%	67%	100%
**Targeting cancer stem cell OXPHOS with tailored ruthenium complexes as a new anti-cancer strategy.**	a. I totally agree	100%	100%	100%		100%	100%
	b. I agree				100%		
	c. Impartial						
	d. I disagree						
	e. I totally disagree						
**A Breast Cancer Stem Active Cobalt (III)-Cyclam Complex Containing Flufenamic Acid with Immunogenic Potential**	a. I totally agree	100%		100%		100%	
	b. I agree		100%		33%		100%
	c. Impartial				67%		
	d. I disagree						
	e. I totally disagree						
**Cobalt (III)-Macrocyclic Scaffolds with Anti-Cancer Stem Cell Activity.**	a. I totally agree	100%	67%	100%	100%	100%	100%
	b. I agree		33%				
	c. Impartial						
	d. I disagree						
	e. I totally disagree	100%					

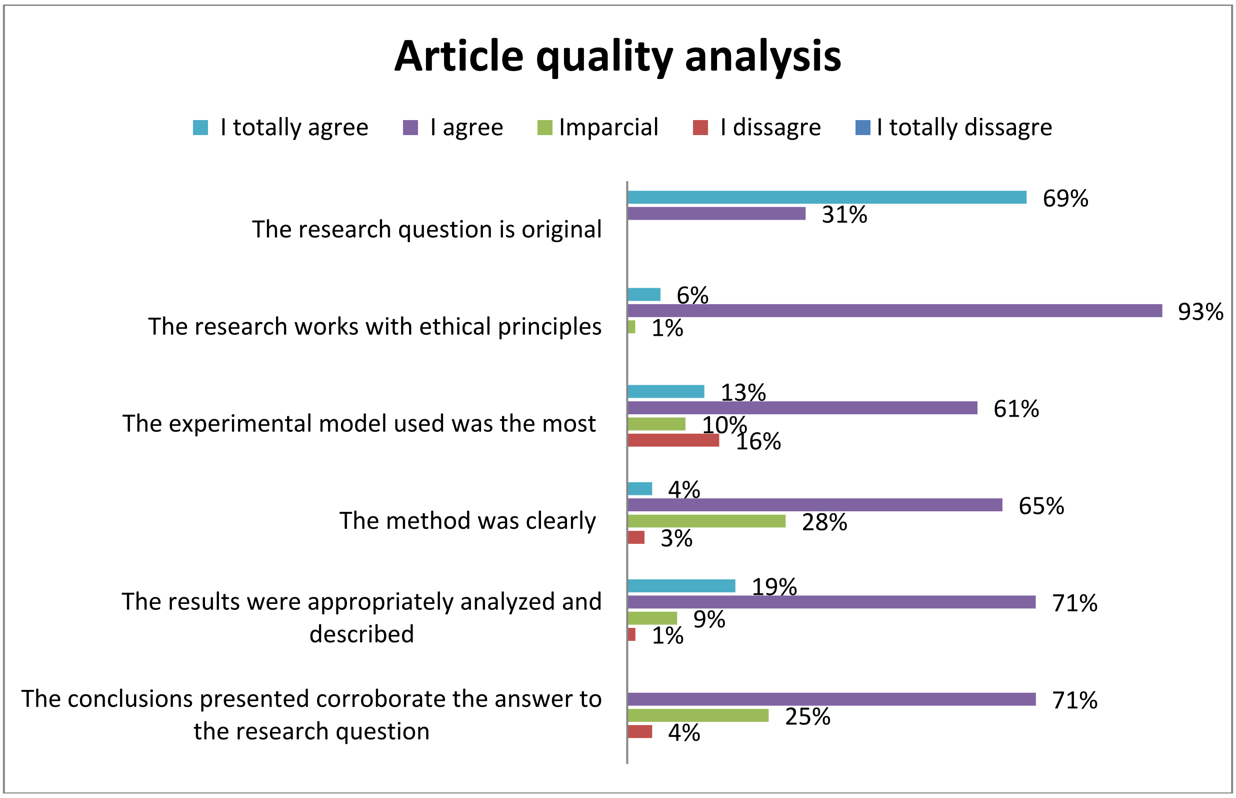

Graph S1: quality analysis of all articles selected for systematic review, grouped by percentage of responses on the Likert scale

Table S3 : Methodologies used in research

First author and year.	Analysis Method
Casangrande, 2024	• Synthesis and characterisation of complexes<br>• X-ray Crystallography<br>• Measurement of Water-Octanol Partition Coefficient (LogP)<br>• Cytotoxicity Studies: MTT Assay<br>• Tumorsphere Formation and Viability Assay<br>• Flow cytometry<br>• TrxR, 20S proteasome and NF-kB activity<br>• Statistical analyses
Li, 2024	• Synthesis and characterisation of complexes<br>• X-ray Crystallography<br>• Measurement of Water-Octanol Partition Coefficient (LogP)<br>• Cytotoxicity Studies: MTT Assay<br>• Tumorsphere Formation and Viability Assay<br>• Cellular Uptake<br>• Intracellular ROS assay.<br>• Immunoblotting analysis.<br>• Annexin V-propidium iodide assay<br>• COX-2 expression assay<br>• CRT cell surface exposure.<br>• ATP assay.<br>• HMGB-1 release.<br>• Phagocytosis assay.
Alcalá, 2024	• Synthesis and characterisation of complexes<br>• Cell lines, primary human PDAC cells, patient samples<br>• Tumorsphere Formation and Viability Assay<br>• Cellular toxicity assay<br>• Colony assay<br>• Flow cytometry and FACS<br>• Zebrafish maintenance and xenograft assays<br>• In vivo toxicity and tumorigenicity assays<br>• RNA sequencing analysis<br>• RNA Preparation and Real-Time PCR<br>• Probabilistic graphical models<br>• Metabolic pathway analyses<br>• Oxygen Consumption Rate (OCR) measurements<br>• Lactate production assay<br>• ATP determination assay<br>• Immunostainings and confocal analysis<br>• Electron microscopy analysis<br>• Western blot analysis<br>• Mitochondrial gradient purification<br>• DNA extraction and PCR<br>• Inductively coupled plasma mass spectrometry (ICP-MS)<br>• Statistical analyses
Fang, 2024	• Synthesis and characterisation of complexes<br>• X-ray Crystallography<br>• Measurement of Water-Octanol Partition Coefficient (LogP)<br>• Cytotoxicity Studies: MTT Assay<br>• Tumorsphere Formation and Viability Assay<br>• Cellular Uptake
Fang, 2023	• Synthesis and characterisation of complexes<br>• X-ray Crystallography

	● Measurement of Water-Octanol Partition Coefficient (LogP)<br>● Cytotoxicity Studies: MTT Assay<br>● Tumorsphere Formation and Viability Assay<br>● Cellular Uptake<br>● Immunoblotting analysis.<br>● Annexin V-propidium iodide assay.<br>● COX-2 expression assay.<br>● CRT cell surface exposure<br>● ATP assay<br>● HMGB-1 release<br>● Phagocytosis assay<br>● Mice.<br>● In vivo studies with 1.<br>● Hematoxylin and eosin staining
Singh, 2023	● Synthesis and characterisation of complexes<br>● Cytotoxicity MTT assay<br>● Mass spectrometry<br>● DCFH-DA assay<br>● Immunoblotting analysis<br>● Tumoursphere formation and viability assay<br>● FITC Annexin V-propidium iodide staining flow cytometry assay
Osei, 2023	● Synthesis and characterisation of complexes<br>● X-ray crystallography<br>● Measurement of water-octanol partition coefficient (LogP<br>● UV-Vis stability studies:<br>● Cell culture<br>● Cytotoxicity MTT assay<br>● Tumoursphere formation and viability assay<br>● Cellular uptake<br>● Intracellular ROS assay<br>● DNA cleavage studies<br>● Immunoblotting analysis<br>● Annexin V-propidium iodide assay
Passeri, 2022	● Synthesis and characterisation of complexes<br>● Measurement of water-octanol partition coefficient (LogP)<br>● Cell Lines and Cell Culture Conditions<br>● Cytotoxicity MTT assay<br>● Osteosphere Formation and Viability Assay
Markova, 2022	● Chemicals<br>● Cell lines and culture conditions<br>● Antiproliferative and phototoxicity testing<br>● Cellular mitochondria colocalization of Ir complex by confocal microscopy<br>● Preparation of CSC-enriched RD.CD133+ cells<br>● 3D cell cultures<br>● FACS characterization of sorted cells<br>● Sphere-forming activity<br>● Mitochondrial mass<br>● Antiproliferative and phototoxic effect of Ir complexes in RD.CD133+ and CD133-spheroids<br>● Cellular accumulation<br>● Distribution of 1 in spheroids<br>● Determination of ROS (CellROX®)<br>● Mitochondrial membrane potential (TMRE)<br>● Mitochondrial ToxGlo™ assay (Promega)<br>● Mitochondrial permeability transition pore assay (BioVision)<br>● Type of cell death (apoptosis/necrosis)

**Johnson, 2022**	● **Synthesis and characterisation of complexes**<br>● **Crystallographic method**<br>● **Electrochemical studies**<br>● **UV-vis absorption spectroelectrochemical studies.**<br>● **Cell culture**<br>● **MTT assay**<br>● **Tumoursphere formation assay**<br>● **Measurement of water-octanol partition coefficient (LogP)**<br>● **Cellular uptake**<br>● **Intracellular ROS Assay.**<br>● **Immunoblotting Analysis.**<br>● **COX-2 Expression Assay.**
**Erkisa, 2021**	● **Chemicals**<br>● **The ATP Viability Assay**<br>● **Detection of Caspase-Cleaved CK18 (M30 Antigen)**<br>● **Determination of Caspase 3/7 Activity and Annexin-V Staining**<br>● **. Induction of Oxidative Stress and ROS Measurement**<br>● **Determination of DNA Damage (ɣH2AX Assay)**<br>● **Statistical Analyses**
**Johnson, 2021**	● **Synthesis and characterisation of complexes**<br>● **Measurement of water-octanol partition coefficient (LogP)**<br>● **Cell Lines and Cell Culture Conditions**<br>● **Tumoursphere formation and viability assay**<br>● **Cytotoxicity MTT assay**<br>● **Cellular uptake**<br>● **Immunoblotting Analysis.**<br>● **Intracellular ROS assay**
**Peng, 2020**	● **Synthetic procedures of Ir1 and Ir2**<br>● **Cell lines and culture conditions**<br>● **Cell viability assay**<br>● **Co-culture of GSC-3# and 293T cells**<br>● **Cell proliferation assay**<br>● **Colocalization assay**<br>● **Cellular uptake mechanism studies**<br>● **Analysis of MMP**<br>● **Measurement of intracellular ROS**<br>● **Soft agar clonal formation assay**
**Acharya, 2020**	● **Syntheses**<br>● **Solution stability and binding study**<br>● **Cell lines and culture condition**<br>● **Cell viability assay**<br>● **Distribution coefficient determination**<br>● **Metal accumulation study in HT-29 cells by ICP-MS and ICP-OES**<br>● **Colonosphere formation assay and drug treatment**<br>● **RNA extraction and cDNA preparation and RT-PCR**<br>● **Immunofluorescence**<br>● **Detection of apoptosis: Annexin-V/PE assay**<br>● **Cell cycle arrest**<br>● **CT-DNA Binding**
**Feld, 2020**	● **Synthesis and characterisation of complexes**<br>● **Measurement of water-octanol partition coefficient (LogP)**<br>● **Cell lines and cell culture conditions**<br>● **Cytotoxicity MTT assay**<br>● **Tumoursphere formation and viability assay**<br>● **Cellular uptake**<br>● **Flow cytometry**
**Xiao, 2020**	● **Synthesis and characterisation of complexes**

	● X-ray Single Crystal Diffraction Analysis.<br>● Measurement of water-octanol partition coefficient (LogP)<br>● Cell Lines and Cell Culture Conditions.<br>● Monolayer Cytotoxicity Studies.<br>● Mammosphere Formation and Viability Assay.<br>● Mammosphere Uptake.<br>● Immunoblotting Analysis.<br>● Ethidium Bromide Displacement Studies.
Novohradsky, 2019	● Isolation of cancer stem cells<br>● Formation of tumorspheres from MCF-7$^{CD44+/CD24-}$ and SKBR-3$^{CD44+/CD24-}$ cells<br>● Viability assay of the mammospheres<br>● Inhibition of the formation of mammospheres from the suspension of MCF-7$^{CD44+/CD24-}$ single cells<br>● Monitoring the changes in the diameter of the preformed MCF-7$^{CD44+/CD24-}$ spheroids<br>● The effect on the heterogeneity of breast cancer cells<br>● Determination of mode of cell death<br>● Propidium iodide (PI) uptake<br>● Immunoblotting experiments
Eskandari, 2019	● Synthesis of the tri-nuclear palladium(II) complex<br>● X-ray Single Crystal Diffraction Analysis.<br>● Measurement of water-octanol partition coefficient (LogP).<br>● Cell Lines and Cell Culture Conditions<br>● Cytotoxicity MTT assay.<br>● Tumorsphere Formation and Viability Assay<br>● Cellular Uptake<br>● Immunoblotting Analysis.<br>● ct-DNA precipitation assay.<br>● Ethidium Bromide Displacement Studies.
Shin, 2019	● Synthesis and characterisation of complexes<br>● Measurement of Water-Octanol Partition Coefficient (Log P)<br>● Cell Lines and Cell Culture Conditions<br>● Cytotoxicity MTT Assay<br>● Tumorsphere Formation and Viability Assay<br>● Cellular Uptake
Zheng, 2019	● Incubation of the complexes with ascorbic acid to generate reduced products.<br>● Treatment of reduced products with 6-carboxy-2',7'-dichlorodihydrofluorescein diacetate (DCFH-DA) to measure reactive oxygen species (ROS) levels.<br>● Use of N-acetylcysteine as a ROS reducing agent.<br>● UV-Vis absorption spectroscopy to identify the formation of copper (I) complexes.<br>● Fluorescence studies to investigate carboxylic acid release from coumarin-3 under reducing conditions.<br>● High-resolution mass spectrometry to identify the main reduced product within CSCs.
Novohradsky, 2019	● Starting materials and reagentes<br>● Physical measurements<br>● Synthesis and characterization<br>● Cell lines<br>● Antiproliferative activity<br>● Cellular uptake<br>● Quantification of platinum bound to DNA<br>● Cell sorting<br>● Generation of spheroids, antiproliferative activity on 3D spheroids

	● Rhabdo/mammosphere formation<br>● Cell death detection<br>● Effect of the treatment on the heterogeneity of the cells<br>● Real-time quantitative PCR
Abe, 2018	● Synthesis and characterisation of complexes<br>● Measurement of water-octanol partition coefficient (LogP)<br>● Cell Lines and Cell Culture Conditions<br>● Tumoursphere formation and viability assay<br>● Cellular uptake<br>● DNA Cleavage Studies.<br>● Immunoblotting Analysis.
Laws, 2018	● Synthesis and characterisation of complexes<br>● Measurement of water-octanol partition coefficient (LogP)<br>● Cell lines and cell culture conditions<br>● Cytotoxicity MTT assay<br>● Tumoursphere formation and viability assay<br>● Cellular uptake<br>● Fluorescence Microscopy<br>● Cytochrome c oxidase Activity<br>● JC-1 Assay<br>● Intracellular ROS Assay<br>● Immunoblotting Analysis.
Lu, 2017	● Synthesis and characterisation of complexes<br>● DNA cleavage and biding studies<br>● UV/VisTritation Studies<br>● Measurement of Water-Octanol Partition Coefficient (Log P)<br>● Cell Lines and Cell Culture Conditions<br>● Cytotoxicity MTT assay<br>● Tumorsphere Formation and Viability Assay<br>● Intracellular ROS assay<br>● Immunoblotting analysis<br>● Flow cytometry
Lu, 2017	● Synthesis and characterisation of complexes<br>● DNA cleavage and biding studies<br>● UV/VisTritation Studies<br>● Measurement of Water-Octanol Partition Coefficient (Log P)<br>● Cell Lines and Cell Culture Conditions<br>● Cytotoxicity MTT assay<br>● Tumorsphere Formation and Viability Assay<br>● Intracellular ROS assay<br>● Immunoblotting analysis<br>● Flow cytometry
Flamme, 2017	● Synthesis and characterisation of complexes<br>● Measurement of water-octanol partition coefficient (LogP)<br>● Cell lines and cell culture conditions<br>● Cytotoxicity MTT assay<br>● Tumoursphere formation and viability assay<br>● Cellular uptake<br>● RNAi signatures<br>● GFP competition assays<br>● Immunoblotting analysis<br>● Propidium iodide (PI) uptake<br>● JC-1 assay<br>● Intracellular ROS assay
Eskandari, 2016	● Synthesis and characterisation of complexes<br>● Measurement of water-octanol partition coefficient (LogP)<br>● Cell lines and cell culture conditions

	Cytotoxicity MTT assay **Tumoursphere formation and viability assay** **Cellular uptake** **RNAi signatures** **GFP competition assays** **Immunoblotting analysis** **Intracellular ROS assay** **Flow Cytometry** **Nanoparticle Encapsulation.** **Nanoparticle Characterisation.** **Payload Release from Nanoparticle.**
**Suntharalingam, 2014**	1. **Cell lines and cell culture conditions**<br>2. **Cytotoxicity MTT assay**<br>3. **Tumoursphere formation and viability assay**<br>4. **Cellular uptake**<br>5. **Fluorescence Microscopy**<br>6. **Intracellular ROS Assay**
